# Chronic cocaine-regulated epigenomic changes in mouse nucleus accumbens

**DOI:** 10.1186/gb-2014-15-4-r65

**Published:** 2014-04-22

**Authors:** Jian Feng, Matthew Wilkinson, Xiaochuan Liu, Immanuel Purushothaman, Deveroux Ferguson, Vincent Vialou, Ian Maze, Ningyi Shao, Pamela Kennedy, JaWook Koo, Caroline Dias, Benjamin Laitman, Victoria Stockman, Quincey LaPlant, Michael E Cahill, Eric J Nestler, Li Shen

**Affiliations:** 1 Fishberg Department of Neuroscience and Friedman Brain Institute, Icahn School of Medicine at Mount Sinai, One Gustave L. Levy Place, Box 1065, New York, NY 10029, USA

## Abstract

**Background:**

Increasing evidence supports a role for altered gene expression in mediating the lasting effects of cocaine on the brain, and recent work has demonstrated the involvement of chromatin modifications in these alterations. However, all such studies to date have been restricted by their reliance on microarray technologies that have intrinsic limitations.

**Results:**

We use next generation sequencing methods, RNA-seq and ChIP-seq for RNA polymerase II and several histone methylation marks, to obtain a more complete view of cocaine-induced changes in gene expression and associated adaptations in numerous modes of chromatin regulation in the mouse nucleus accumbens, a key brain reward region. We demonstrate an unexpectedly large number of pre-mRNA splicing alterations in response to repeated cocaine treatment. In addition, we identify combinations of chromatin changes, or signatures, that correlate with cocaine-dependent regulation of gene expression, including those involving pre-mRNA alternative splicing. Through bioinformatic prediction and biological validation, we identify one particular splicing factor, A2BP1(Rbfox1/Fox-1), which is enriched at genes that display certain chromatin signatures and contributes to drug-induced behavioral abnormalities. Together, this delineation of the cocaine-induced epigenome in the nucleus accumbens reveals several novel modes of regulation by which cocaine alters the brain.

**Conclusions:**

We establish combinatorial chromatin and transcriptional profiles in mouse nucleus accumbens after repeated cocaine treatment. These results serve as an important resource for the field and provide a template for the analysis of other systems to reveal new transcriptional and epigenetic mechanisms of neuronal regulation.

## Background

Alterations in gene expression contribute importantly to the long-lasting changes that drugs of abuse induce in the brain’s reward circuitry [1]. Numerous studies to date have utilized gene expression microarrays to obtain an unbiased view of such alterations, and several transcription factors have been implicated in mediating some of these effects. Moreover, several target genes discovered with these approaches have been directly implicated in the complex cellular and behavioral plasticity induced in this reward circuitry associated with drug addiction. However, relatively little information is yet available concerning the detailed molecular steps through which such alterations in gene expression are induced, and available information is limited by the reliance to date on microarray technology.

Recently, epigenetic regulation, such as multiple histone modifications and DNA methylation, has emerged as a key mechanism of addiction-related phenomena [2-6]. Drugs of abuse such as cocaine have been shown to alter the expression levels of several histone- and DNA-modifying enzymes within key brain reward regions, such as the nucleus accumbens (NAc) [7-10]. Importantly, these enzyme changes, which include altered levels of certain histone deacetylases and histone lysine methyltransferases, are associated with cocaine-induced changes in histone acetylation or lysine methylation at many specific candidate genes, which are already known to be involved in cocaine action [9,11]. Recently, cross-talk has been demonstrated between regulation of histone acetylation and lysine methylation in NAc [12]. While many gene-specific histone changes are in a direction commensurate with the altered enzyme expression levels, a large subset of observed changes are in the opposite direction, which further underscores the complexity of chromatin regulation *in vivo*.

To extend these candidate gene studies, we recently mapped cocaine-induced changes in the genome-wide binding of pan-acetylated H3, pan-acetylated H4, and dimethylated H3 (at both K9 and K27) in NAc by use of ChIP-chip assays (chromatin immunoprecipitation followed by promoter microarrays) [13]. This study identified hundreds of novel gene targets of cocaine, but was inherently limited in several important ways. First, ChIP-chip by design restricts analysis to proximal promoter regions of genes only, even though we know that much chromatin regulation occurs in other genic, as well as intergenic, regions. Second, recent evidence indicates that net levels of gene transcription result from the complex interplay of large numbers of chromatin modifications, which act in concert in ways that remain incompletely understood [14,15]. Third, genome-wide characterizations of gene expression in brain have to date relied mainly on microarrays, as opposed to RNA-seq, which provides unprecedented advantages such as more precise measurement of levels of transcripts and their splicing isoforms [16]. Finally, recent evidence from *in vitro* non-nervous tissues has suggested that alternative splicing is regulated by chromatin modifications at specific genes [17]. As alternative splicing is a process by which pre-mRNAs are differentially spliced, and lead to the expression of several mRNAs from a single gene, it provides an essential mechanism that expands and diversifies the proteome [18]. However, little is known about the contribution of alternative splicing to cocaine action or how it is influenced by epigenetic regulation in brain.

To address these limitations, we carried out a more comprehensive analysis of the cocaine-induced transcriptome and epigenome in the mouse NAc. We used ChIP-seq (ChIP followed by next-generation sequencing), which offers several advantages over ChIP-chip [19], to characterize numerous chromatin modifications within this brain region in response to repeated cocaine administration. We focused on several transactivation marks (H3K4me1, H3K4me3, and H3K36me3) and repression marks (H3K9me2, H3K9me3, and H3K27me3). These histone modifications were selected to cover enhancer (H3K4me1), promoter (H3K4me3, H3K27me3), gene body (H3K36me3), and intergenic (H3K9me2, H3K9me3) regions [20,21]. We also analyzed binding of RNA polymerase II (RNA pol II). These ChIP-seq data were then overlaid onto RNA-seq data to capture cocaine-induced changes in gene expression, including those resulting from regulation of pre-mRNA alternative splicing.

Our findings identify many chromatin signatures - unique combinations of histone modifications that predict cocaine regulation of gene expression, a large portion of which is mediated by previously uncharacterized changes in alternative splicing. The robustness of this epigenomic analysis is further demonstrated by its ability to predict the involvement of a novel splicing factor, termed A2BP1 (also known as RBFOX1 or FOX-1), in cocaine action.

## Results

### Cocaine-regulated transcriptomic changes in mouse nucleus accumbens

To characterize the transcriptome of mouse NAc, we used RNA-seq to measure the expression levels of all polyA containing transcripts in NAc of mice treated chronically with cocaine or saline (control); we used a standard treatment regimen of daily 20 mg/kg intraperitoneal doses of cocaine for 7 days with animals analyzed 24 h after the last dose, a procedure known to induce numerous, highly validated molecular and cellular adaptations to the drug [9]. This procedure is also behaviorally relevant, as it induced locomotor sensitization, an extensively validated form of behavioral plasticity to repeated cocaine administration (Additional file 1). To account for inter-animal variations, we obtained three biological replicates for each treatment group, with each replicate representing NAc pooled from five animals. Samples were sequenced by an Illumina HiSeq2000 machine. We obtained 93 to 97 million short reads of 100 bp from each replicate. Of these, 65 to 67% were successfully aligned to a reference gene database (Ensembl: Mus musculus, NCBIM37.62) by TopHat [22]. The quality of the data were assessed by the RNA-SeQC package [23], which revealed that approximately 95% and 81% of the mapped reads are intragenic and exonic, respectively, and that the sequencing data are not overrepresented by mitochondrial reads (Additional file 2). Overall, our aligned short reads represent 21,892,637,222 and 21,717,236,397 transcribed nucleotides for the mouse NAc transcriptome under cocaine and saline treatment, respectively. These data are sufficient to provide on average of approximately 183× coverage for mouse exomes under both conditions.

We used the Cufflinks package [24] to perform differential analysis for changes in gene expression. For our initial analysis, we used stringent false discovery rate (FDR) cutoffs of <10%, fold change >1.25, and Reads Per Kilobase transcript per Million reads (RPKM) >1, and identified 92 genes (61 increased, 31 decreased; Additional file 3) that are differentially expressed in NAc after repeated cocaine administration (see Materials and methods). To confirm that the expression changes identified reflect the actions of repeated, not acute, cocaine treatment, we performed RNA-seq on NAc obtained from mice treated with a single dose of cocaine, with animals analyzed 24 h later. The data were analyzed the same way and passed all quality assessments mentioned above (Additional file 2). We identified 55 genes (42 increased, 13 decreased; Additional file 3) that are differentially expressed in NAc in response to a single cocaine dose, only 4 of which overlapped with the chronic cocaine-regulated genes. In addition, two of the four genes showed the opposite direction of regulation. We therefore conclude that the vast majority of gene expression changes induced by repeated cocaine are very different from those induced by acute cocaine.

**Figure 1:**
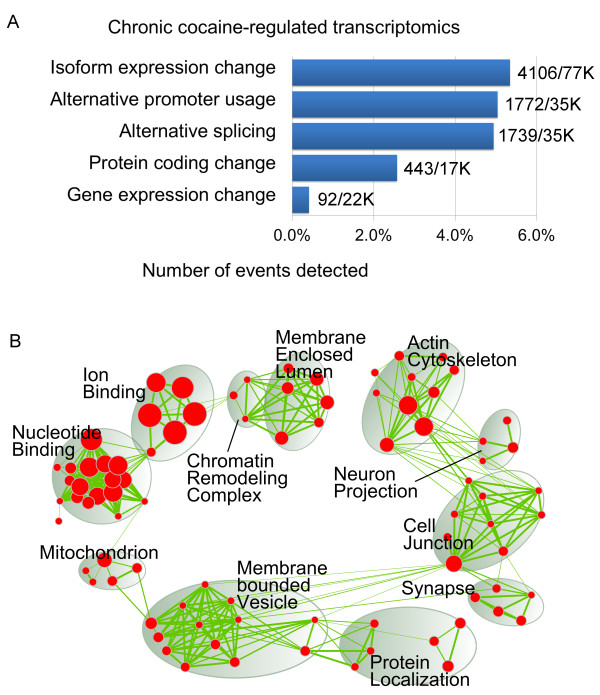
**Chronic cocaine-induced transcriptome in mouse NAc. (A)** Cocaine-regulated transcriptomic events versus total events illustrated as percentages (for example, 92 out of 22,000 genes show significant expression changes), which are based on Cuffdiff prediction (see Materials and methods). **(B)** Gene Ontology (GO) enrichment for differentially spliced genes is illustrated as an enrichment map [26]: each node represents an enriched GO term and its size represents the number of genes; the color intensity of the node represents the statistical significance; the strength of the green lines represents the number of common genes shared between two nodes; the GO terms that are clustered together are circled and summarized with a name. Singletons are not shown; a full list of enriched GO terms is in Additional file 6.

**Figure 2:**
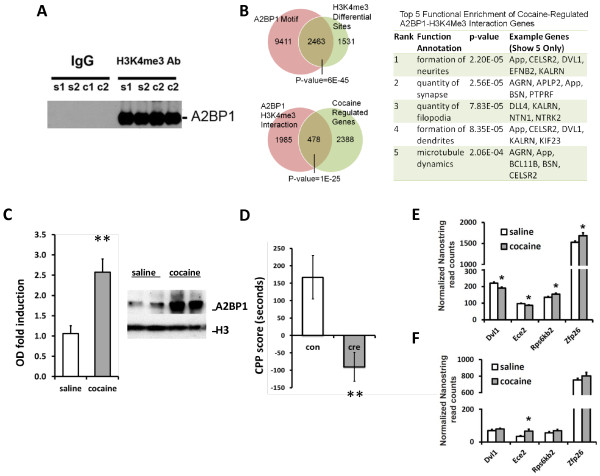
**Role of A2BP1 in cocaine responses. (A)** Co-immunoprecipitation shows A2BP1 western blotting of H3K4me3 or IgG pulldown of whole NAc lysate. Two repeated cocaine-treated samples and two saline control samples are shown. **(B)** The two Venn diagrams show overlap between: A2BP1-motif containing genes versus H3K4me3 differential site-containing genes (upper); A2BP1 motif- and H3K4me3 differential site-containing genes versus cocaine-regulated differential and spliced genes (lower). The table shows the top five enriched functional terms of the 478 overlapped genes from the lower Venn diagram. **(C)** Western blot analysis of A2BP1 in nuclear lysates of NAc after repeated cocaine. Error bars are mean ± standard error of the mean (SEM) derived from eight cocaine treated and eight saline treated control mice, respectively. **(D)** Conditioned place preference (CPP) scores of AAV-Cre-GFP-injected *A2bp1*^*loxP/loxP*^ mice (cre) with AAV-GFP-injected *A2bp1*^*loxP/loxP*^ mice as control (con). Error bars are mean ± SEM derived from seven cre and eight control samples. **(E)** Four predicted A2BP1 target candidate genes were chosen for Nanostring validation. All show the same direction of transcription change after chronic cocaine treatment as observed in RNA-seq. Error bars are mean ± SEM derived from 14 cocaine and 14 saline treated samples. **(F)** Cocaine-induced transcription change as observed in **(E)** are lost (*Rps6kb2*, *Zfp26*, *Dvl1*) or in one case reversed (*Ece2*) when *A2bp1* was conditionally knocked down by using AAV-Cre viral injection in NAc. Error bars are mean ± SEM derived from five cocaine and six saline treated samples. *P < 0.05, **P < 0.01.

On average, each protein coding gene in the reference database encodes 3.4 transcripts. For a coding gene, the transcripts may share the same transcriptional start site (TSS; that is, alternative splicing) or have different TSSs (that is, alternative promoter usage) [25]. Interestingly, our analysis revealed that promoter usage and splicing changes are much more widespread than differential expression in response to repeated cocaine. For this analysis, we used Cufflinks with an FDR cutoff of only <10% . Although this approach would be expected to increase the number of false positive hits, it also reduces the number of false negative hits, which is important for our subsequent ability to overlay these RNA-seq data with epigenetic modifications (see below). This alternative analysis identified 1,772 and 1,739 TSS groups to have alternative promoter usage and alternative splicing (Additional file 3), respectively, comprising approximately 5% of all TSS groups (Figure 1A). Looking at individual transcripts, we also found 4,129 to be differentially expressed (Additional file 3), comprising approximately 5% of all coding transcripts. A few examples of alternatively spliced genes are shown in Additional file 4. We validated these RNA-seq data by confirming altered expression of key transcripts in independent tissue samples by use of Nanostring technology (Figure 2E; Additional file 5).

Combining the genes that show chronic cocaine-induced changes in alternative promoter usage or alternative splicing yields 2,998 genes that are differentially spliced, which represent 35% of all differentially expressed genes. To understand the functional roles of these differentially spliced genes, we performed gene ontology (GO) analysis [27,28] and identified 110 (FDR <10%, Fisher’s exact test) enriched GO terms (Additional file 6). We created an enrichment map [26] (Figure 1B) to represent these functional terms and found that the differentially spliced genes are associated with very diverse functions and cellular components. With respect to molecular functions, two major groups are involved in nucleotide binding and ion binding, with one minor group participating in protein localization. In terms of cellular components, four major groups are involved in membrane enclosed lumen, actin cytoskeleton, cell junction, and membrane bounded vesicle, with four minor groups involving chromatin remodeling complex, synapse, neuron projection, and mitochondrion. These results suggest that differentially transcribed or spliced genes play substantial roles in the transcriptional perturbations induced in NAc by chronic cocaine.

### Cocaine-regulated epigenomic changes in mouse nucleus accumbens

The lasting behavioral abnormalities induced by chronic cocaine treatment have been attributed, in part, to epigenomic changes involving post-translational modifications to histone tails [2,29]. We thus chose six histone modifications (H3K4me1, H3K4me3, H3K9me2, H3K9me3, H3K27me3, and H3K36me3), as well as total RNA pol II, to investigate the epigenomic changes induced in mouse NAc by repeated cocaine exposure. They were chosen to ensure coverage of gene promoters, gene bodies, enhancers, as well as intergenic regions and to reflect mechanisms of gene activation and repression (see Background). We used three biological replicates for each mark, with each replicate again representing tissue pooled from five animals. After mapping the reads to the mouse reference genome, we removed the ones that are redundant at the same location and strand (Additional file 7). We thereby obtained uniquely aligned, non-redundant reads with a total number varying from 8 to 465 million for each of the seven marks under each condition (Additional file 8). Overall, these ChIP-seq data represent a highly informative collection of histone modifications and RNA pol II enrichment, comprising total read counts of 1,105,314,297 and 1,114,544,836 (Additional file 8) under cocaine and saline treatment, respectively.

**Figure 3:**
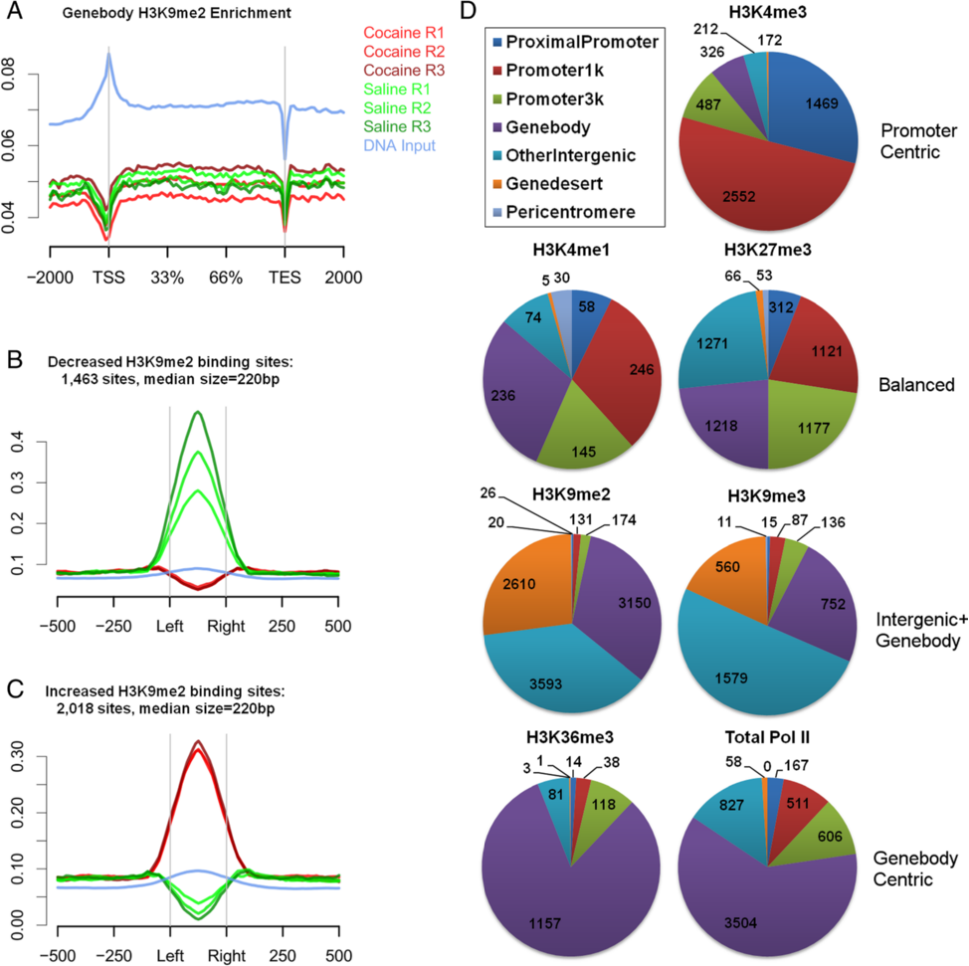
**Repeated cocaine-regulated epigenome measured by ChIP-seq. (A-C)** Averaged coverage plots of different biological replicates of H3K9me2 and DNA input for different genomic regions. The x-axis represents the genomic region from 5’ to 3’. The y-axis represents coverage that has been normalized to the number of aligned reads per million mapped reads (RPM). **(A)** Genebody of the entire mouse genome. **(B)** Differential sites that show decreased H3K9me2 binding in genic regions. **(C)** Differential sites that show increased H3K9me2 binding in genic regions. **(D)** Pie charts show the genome-wide distribution patterns of the differential sites of the seven chromatin marks.

To determine the enrichment for the 42 (7 marks × 2 conditions × 3 replicates) ChIP-seq samples at genic regions, we generated plots (Figure S4A-G in Additional file 9) at TSSs, genebodies, and transcriptional end sites using a program called ngs.plot [30]. The enrichment patterns for the seven marks from our study are highly similar with those generated in cell culture studies [21,31], indicating that the ChIP-seq data are of good quality and that *in vivo* brain histone modifications share similar gross distribution patterns with other tissues. Cocaine does not cause significant changes in any of these gross distribution patterns (Figure S4A-G in Additional file 9). We therefore presumed that cocaine-induced changes are localized to specific genomic sites. To reveal the location-specific changes for each of the seven marks, we used diffReps [32] to identify the chromatin modification sites (termed differential sites) that show significant differences between repeated cocaine and saline conditions. We identified thousands to tens of thousands of differential sites (ranging from 200 bp to 1,200 bp in size) for the various marks (FDR <10%; Figure S4H in Additional file 9; Additional file 10). Together, these differential sites represent a massive amount of epigenomic changes induced in mouse NAc by cocaine. Further examination revealed appreciable changes in the enrichment of most of these marks at differential sites (Figure S4A-G in Additional file 9). For example, H3K9me2 displays significant dynamics at its differential sites, with robust increases or decreases (Figure 3B,C) induced by cocaine despite the fact that it shows a 'flat-line' like pattern of enrichment at the genebody level genome-wide (Figure 3A). This further strengthens our notion that cocaine-regulated chromatin changes are highly localized and may correspond to specific functions.

e classified all differential sites into several categories based on their genomic locations (Figure 3D) and found that the seven marks can generally be divided into the following groups: 'promoter-centric' (H3K4me3), 'balanced' (H3K4me1 and H3K27me3), 'genebody-centric' (H3K36me3 and RNA pol II), and 'genebody + intergenic' (H3K9me2 and H3K9me3). The distributions of the differential sites are very similar to that of basal peaks (Additional file 11) for these marks. To further elucidate the potential functions of these chromatin changes, we performed molecular pathway analysis through IPA (Ingenuity Systems) [33] for the genes that are associated with the differential sites. In total, 248 canonical pathways were found to be enriched (*P* < 0.05, Fisher’s exact test). To identify the most important pathways, we ranked them by co-occurrence score among the seven marks and examined the top 30 (Additional files 12 and 13). We found that many of the top ranked pathways have been previously implicated in drug addiction, for example, 'CREB signaling in neurons' (top 2, score = 37.5), 'axonal guidance signaling' (top 7, score = 28.2), 'synaptic long term potentiation' (top 8, score = 27.8), and 'WNT/β-catenin signaling' (top 9, score = 26.5). In addition to the canonical pathways, we created three customized gene lists to represent additional knowledge of addiction pathophysiology and also found them to be enriched: 'actin cytoskeleton' (score = 25.0); 'synaptic plasticity' (score = 17.3); and 'growth factors' (score = 2.2). This further demonstrates that the chromatin changes we identified are highly specific to brain functions that contribute to addiction. Interestingly, the two marks that are associated with gene activation, H3K4me3 and RNA pol II, show much more pronounced enrichment than the other five marks among the top 30 pathways (Additional files 12 and 13).

### Chromatin signatures associated with pre-mRNA alternative splicing

We next determined that the chromatin differential sites show sharp proximity to exons within a 10 kb window (Additional file 14), suggesting a possible role for the seven marks in cocaine-mediated pre-mRNA alternative splicing. Recent *in vitro* investigations showed that the splicing of exons into a mature mRNA occurs co-transcriptionally [17]. Previous studies have also demonstrated that some histone marks can act as beacons in exon definition [34,35] or play important roles in recruiting splicing factors to pre-mRNAs [36,37]. We found in our dataset that all seven marks show local enrichment at exons to varying degrees and are correlated with transcriptional levels under basal conditions (Additional file 15). More specifically, exonic enrichments of H3K4me1, H3K4me3, H3K36me3, and total RNA pol II are positively correlated with transcriptional levels; that of H3K9me2, H3K9me3, and H3K27me3 are negatively correlated.

Despite these correlations, the interplay between histone marks and splicing regulators is complicated. For example, the repressive mark H3K9me3 has recently been found to facilitate the inclusion of variant exons of several genes via a mechanism that involves decreased RNA pol II elongation rate [38]. Another study [37] investigated the roles of several histone marks in selection of two mutually exclusive exons between two human cell lines, and found that two groups (H3K27me3, H3K4me3, and H3K9me1 versus H3K36me3 and H3K4me1) of histone marks regulate the two exons in the opposite direction. However, how extensive and how exactly the histone modifications influence alternative splicing *in vivo*, especially under pathological conditions (for example, after chronic cocaine exposure), is unknown.

RNA-seq provides unique advantages for alternative splicing analysis [16]. Some programs achieve this goal by looking at the read counts of individual exons [39,40]. However, transcripts often share common exons whose read counts thus convey nothing unique about each transcript’s expression levels. On the other hand, each transcript must contain unique exonic regions, which provide information about the transcript’s expression level. The program we used - Cufflinks - assigns reads proportionally to each individual transcript by solving an optimization problem on the unique exonic read counts [24,25]. In line with this approach, we needed a method to describe the chromatin changes associated with each transcript. We therefore devised a systematic approach called 'chromatin signature' that allowed us to profile the epigenomic changes associated with each transcript in a unified fashion (Additional files 7 and 16). In this analysis, it was important to use our broader Cufflinks evaluation of differential transcriptional changes, since it reduces false negative discovery rates, while overlaying such data with multiple chromatin endpoints achieves the higher stringency needed to reduce false positive discovery.

Briefly, we first classified all exons into six different types (Additional file 16): 'promoter', 'canonical', 'variant', 'alternative acceptor', 'alternative donor', and 'polyA'. Each exon type represents a unique combination of exon-intron boundaries. We also derived the neighboring intronic regions (150 bp) for each exon, which are also implicated in splicing regulation [41]. In total, we defined 335,779 and 441,648 unique exonic and intronic regions on the genome and calculated each mark’s enrichment difference between cocaine and saline at each region (Additional file 7). In addition, we included the 500 bp intergenic regions upstream of the TSS and the enhancers defined by H3K4me1 peaks (Additional file 7) to complement a chromatin signature. We removed the shared regions (that is, canonical exons and introns at canonical acceptor and donor sites) from consideration as they do not convey distinct chromatin information between transcripts. This results in 13 different types of regions (Additional file 16) upon which chromatin modifications are defined for a transcript. Based on the resulting chromatin signatures, we constructed a matrix of approximately 77,000 (coding transcripts) × 91 (7 marks × 13 genomic regions; Additional files 7 and 16) to represent the splicing-related chromatin modifications mediated by cocaine for the entire mouse transcriptome.

**Figure 4:**
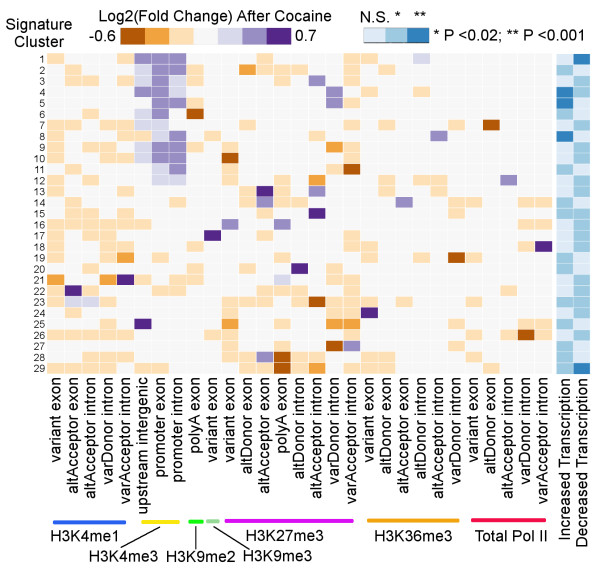
**Heatmap of cocaine-regulated chromatin modifications of the 29 signature clusters that associate with transcriptional regulation.** In the left panel, each row represents a cluster and each column represents an epigenomic mark at a specific genomic region; each square represents the averaged chromatin modification change in log2 scale; purple and orange colors indicate increased or decreased binding and the darkness indicates the magnitude of change. The heatmap in the right panel illustrates the statistical significance of each cluster’s association with transcriptional change. N.S., not significant.

We first tested whether those chromatin signatures can be grouped by similar patterns and whether they can be associated with transcriptional changes. To reduce the complexity, we performed genome-wide association (Additional files 7 and 17) to remove the transcripts that show no detectable chromatin changes. We also removed mark-region combinations that show little correlation with transcriptional change. Interestingly, we did not find correlations for the chromatin modifications at the 24,745 H3K4me1-labeled distal (more than ±1 kb from the TSS) enhancers and their corresponding target transcripts’ expression change (Additional file 17). We therefore removed the enhancer regions from further analysis. We also found 41 mark-region combinations to be not significant. After this filtering, we obtained a smaller matrix of approximately 33,000 × 43 and then performed K-means clustering to identify (Additional file 7) clusters of co-expressed chromatin signatures. We found 29 clusters (Figure 4; *P* < 0.02, Fisher’s exact test) to be significantly associated with transcriptional change. Each cluster represents a combination of enrichment differences of the seven marks (Additional file 18) that lead to increased or decreased transcript levels (Figure 4). For example, the H3K4me3 generally shows increased binding around TSSs for clusters 1 to 12. However, depending on the combination with other histone marks, clusters 1 to 12 show either increased or decreased transcription (Figure 4). H3K4me1 overall shows decreased binding at variant and alternative acceptor exons, including neighboring intronic regions across all 29 clusters. H3K27me3 displays the most dynamics among the seven marks for the 29 clusters, while H3K9me2 and H3K9me3 only regulate polyA and variant exons, respectively. Notably, most of the marks show regulation at the intronic regions, indicating the importance of introns in determining splicing.

**Figure 5:**
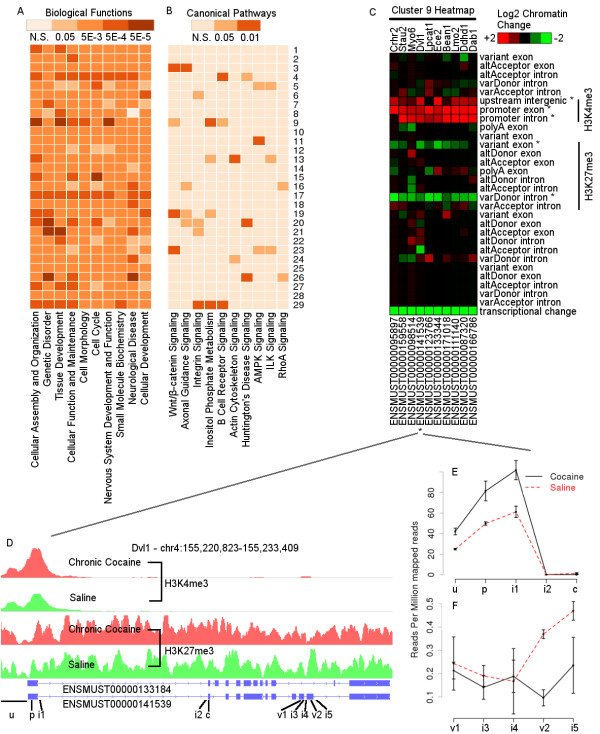
**The functional enrichment of the 29 signature clusters and identification of significant chromatin modification regions for an example cluster. (A,B)** Each row represents a cluster and each column represents a functional term or canonical pathway. The color of each square represents the statistical significance of enrichment. The enriched terms are ranked by co-occurrence and only the top 10 are shown. **(A)** Biological functions. **(B)** Canonical pathways. N.S., not significant. **(C)** The chromatin modification heatmap for cluster 9. Each column represents a transcript and each row represents a chromatin mark at a specific genomic region. Only 10 example transcripts are shown here. Each square represents the chromatin change in log2 scale. The transcriptional change is simply indicated as a binary value. The significantly modified regions are marked by asterisks to the right of their names. An example transcript, ENSMUST00000141539 of gene *Dvl1* (a protein in the WNT/β-catenin pathway), is further illustrated in **(D-F)**. **(D)** Genome browser screenshot for the demo transcript with H3K4me3 and H3K27me3 in the top and bottom two tracks, respectively. The exon structures of the demo transcript and another transcript of the same gene are shown below the chromatin tracks. The genomic regions are marked by letters ('u', upstream intergenic; 'p', promoter exon; 'c', canonical exon; 'v', variant exon; 'i', intron), and are further followed by numbers to distinguish different regions. **(E,F)** Mean and standard error of the mean of the significant regions with some neighbors are shown as line plots with error bars. The x-axis represents different regions from 5’ to 3’ and the y-axis represents normalized coverage. **(E)** H3K4me3 at u, p, i1, i2 and c. **(F)** H3K27me3 at v1, i3, i4, v2 and i5.

Functional enrichment analysis through IPA for the 29 signature clusters identified 90 (*P* < 0.05, Fisher’s exact test; Additional file 19) enriched biological function terms. The top 10 functional terms (Figure 5A) based on co-occurrence score are specific to the nervous system and neurological diseases or related to some essential cellular or molecular process. Interestingly, the top ranked biological functions seem to be ubiquitously enriched among all signature clusters. On the contrary, the 348 (*P* < 0.05, Fisher’s exact test; Additional file 19) enriched canonical pathways are more specific for each cluster (Figure 5B, top 10 are shown), for instance, 'WNT/β-catenin signaling', 'axon guidance signaling', and 'actin cytoskeleton signaling'. This suggests that cocaine-induced chromatin signature changes are not random and have functional implications in drug responses. Furthermore, this indicates that cocaine-induced transcriptional changes within the same pathway may share common chromatin signatures, which is characterized by unique combinatorial histone modification patterns.

### Chromatin signature-associated protein regulators

We next used these chromatin signatures to infer the types of chromatin-associated proteins that may convert the epigenetic information into transcriptional change. The 29 signature clusters we identified represent groups of transcripts that share common chromatin modification patterns. This co-expression indicates that each of the clusters is co-regulated by a few common protein regulators that may interact with chromatin during transcription. We focused on transcription factors and splicing factors that may be involved in this process. To illustrate our approach, an example is given in Figure 5C-F. Briefly, the region-mark combinations that show significant chromatin change (*P* < 1E-10, one group *t*-test) in the same direction are first identified from each cluster. Each transcript is then analyzed and only the regions that show a significant chromatin binding difference (*P* < 0.05, one-tailed Student’s *t*-test) between cocaine and saline are picked for further analysis. The sequences of the same type of region (such as variant exon) from the same cluster are pooled and motif analysis (Additional files 7 and 20) by MEME [42] is performed. In this manner, we found 32 and 58 uniquely identifiable motifs from the intragenic and intergenic regions, representing potential splicing and transcription factors that control the 29 clusters, respectively.

**Figure 6:**
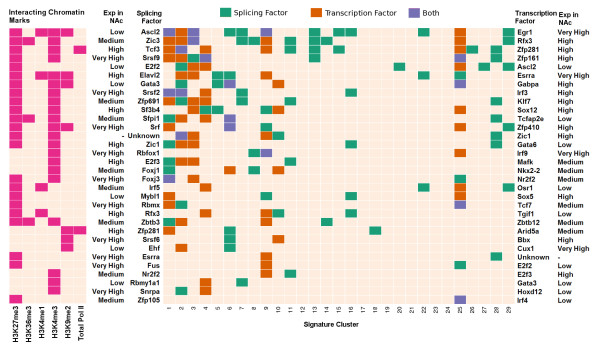
**Splicing and transcription factors inferred from the 29 signature clusters induced by repeated cocaine and their interacting epigenomic marks.** Splicing and transcription factors are indicated on the right panel by different colors. Green means the enrichment of a splicing factor on the left; red means the enrichment of a transcription factor on the right; blue means the enrichment of both. All 32 splicing factors and the top 32 transcription factors are shown here. Each column represents a signature cluster. The gene expression level for the regulators is labeled as 'Very High' (RPKM >20), 'High' (RPKM >5 and ≤20), 'Medium' (RPKM >1 and ≤5), and 'Low' (RPKM ≤1) based on our RNA-seq data. The chromatin marks that interact with the splicing factors are shown on the left panel by pink color.

The splicing and transcription factors are ranked by co-occurrence and illustrated as a heatmap (Figure 6, right panel). Numerous splicing factors identified here have been reported to have functions in neuronal differentiation, neurological diseases, or synaptic plasticity, such as ELAVL2 [43,44], ZIC1 [45], A2BP1 (official gene symbol *Rbfox1*) [46], and FUS [47,48]. Interestingly, two E2F family proteins, E2F2 and E2F3, are enriched both as splicing factors and transcription factors. The E2F family proteins have been implicated in neurogenesis [49] and are known to interact with SIRT1 [50], a histone deacetylase that has been implicated in cocaine regulation [13,51]. Among transcription factors, the top ranked EGR1 protein is enriched in five clusters and is known to modulate synaptic plasticity in part through the direct regulation of its target genes [52]. Notably, only one splicing/transcription factor, ZIC1, shows differential expression in our RNA-seq data, suggesting that cocaine mainly regulates the target genes via their interaction with perturbed chromatin modifications at the target loci. The epigenomic marks that interact with the splicing factors are shown as a heatmap (Figure 6, left panel), where H3K27me3 and H3K4me3 display a dominant presence. The only mark that interacts with transcription factors at upstream intergenic regions is H3K4me3, which shows increased enrichment after cocaine.

### A2BP1 is an important regulator of cocaine responses

As noted above, our motif analysis identified A2BP1 as a potentially important splicing factor that regulates clusters 8 and 9 (Figure 6). A2BP1 belongs to a family of RNA binding proteins that is composed of two other homolog splicing factors, RBFOX2 (RBM9 or FOX-2) and RBFOX3 (HRNBP3, NEUN, or FOX-3). Human A2BP1 was first identified through its interaction with Ataxin-2, the protein mutated in spinocerebellar ataxia type II [53]. Mutations in the human *A2BP1* gene have since been associated with several other neurological syndromes, including mental retardation, epilepsy, and autism spectrum disorders [54-57]. Recent studies also implicate A2BP1 in regulating neuronal excitability as well as neuronal adaptations to stress [58,59]. Our RNA-seq data demonstrated that *A2bp1* is highly expressed in mouse NAc (RPKM = 90, >97% of the genome in NAc). By binding to the CAUGCA motif, A2BP1 controls many neuronally regulated exons [60]. Indeed, some A2BP1-dependent alternative exons have already shown dysregulated splicing in human autism spectrum disorders [61].

In clusters 8 and 9 (Figure 6), the site of discovery for the A2BP1 motif is located in promoter exons where H3K4me3 shows increased binding after cocaine. This indicates an interaction between this splicing factor and the histone tail modification, which has not to date been documented. We first validated such H3K4me3 enrichment at selected loci from clusters 8 and 9 with ChIP-quantitative PCR (Additional file 21). Next, we experimentally examined the physical binding between the two molecules. A co-immunoprecipitation assay demonstrated a significant enrichment of A2BP1 in the H3K4me3 immunoprecipitation pulldown from NAc extracts (Figure 2A). Notably, this binding between A2BP1 and H3K4me3 appeared to be specific, since no A2BP1 was detected in the IgG pulldown control samples.

We then performed a genome-wide scan [62] for the A2BP1 motif obtained from our analysis on the regions where the chromatin signatures were defined in this study, and found 37,993 hits (motif match *P* < 1E-4). We further intersected the genes whose exons or introns contain a predicted A2BP1 binding motif (n = 11,874) with the genes that contain H3K4me3 differential sites (n = 3,994) and found the overlap (n = 2,463) to be statistically significant (Figure 2B; *P* = 6E-45, Fisher’s exact test). This finding further strengthened the enrichment of A2BP1 at cocaine-regulated H3K4me3 binding sites. Moreover, the genes that display a significant A2BP1 and H3K4me3 interaction (n = 2,463) also show substantial overlap (Figure 2B; *P* = 1E-25, Fisher’s exact test) with cocaine-regulated genes (n = 2,866), including those displaying differential expression or alternative splicing. IPA analysis of the 478 cocaine-regulated, A2BP1-H3K4Me3 interaction genes (Figure 2B; Additional file 22) revealed 174 functional terms to be enriched (*P* = 0.05, Fisher’s exact test; Additional file 23), with the top five terms (Figure 2B) relating to neurite formation and synapse dynamics.

Though our RNA-seq data did not show significant cocaine regulation of *A2bp1*’s mRNA levels, we used western blotting to test whether chronic cocaine treatment alters A2BP1 at the protein level in NAc. Consistent with the mRNA finding, we did not observe a significant change of A2BP1 from whole NAc lysates (data not shown). However, chronic cocaine induced a >2.5-fold increase in A2BP1 protein levels in nuclear lysates (Figure 2C). This nucleus relocation is consistent with previous findings from cultured neurons that depolarization induces nuclear migration of A2BP1, which increases the splicing of A2BP1 target genes [60].

To gain further insight into the functional importance of A2BP1 in behavioral responses to cocaine, we carried out conditioned place preference (CPP) assays in mice with a local knockout of *A2bp1* from NAc. CPP provides an indirect measure of drug reward. Adult floxed *A2bp1* (*A2bp1*^*loxP/loxP*^) mice [58] were injected intra-NAc with an adeno-associated virus (AAV) vector expressing Cre-GFP or GFP alone. Though AAV-GFP-injected *A2bp1*^*loxP/loxP*^ mice developed a significant cocaine preference at a moderate drug dose (7.5 mg/kg), AAV-Cre-GFP-injected *A2bp1*^*loxP/loxP*^ mice displayed no place conditioning (Figure 2D). Thus, knockdown of *A2bp1* in NAc decreased the rewarding effects of cocaine.

Lastly, we selected representative predicted A2BP1target genes within clusters 8 and 9 and tested whether conditional *A2bp1* knockdown in NAc affects their expression. Consistent with our RNA-seq data, by use of Nanostring validation with independent tissue samples, we confirmed increased expression of *Rps6kb2* and *Zfp26*, as well as decreased expression of *Dvl1* and *Ece2* (Figure 2E). Importantly, all of these chronic cocaine-triggered expression changes were lost when *A2bp1* was conditionally knocked down in NAc; in fact, *Ece2* displayed cocaine regulation in the opposite direction in the absence of *A2bp1* (Figure 2F). These findings further support the importance of this splicing factor in cocaine action as inferred from our bioinformatic analyses.

## Conclusion and discussion

Results of the present study provide the most complete profiling to date of the cocaine-induced transcriptome and epigenome in NAc. We defined the binding patterns of six histone modifications and of RNA pol II genome-wide under repeated cocaine and saline conditions and correlated these patterns with the repeated cocaine-induced transcriptome. We show that different histone modifications act in a combinational fashion to create chromatin signatures that correlate with altered gene expression and, more specifically, with dramatic cocaine regulation of alternative splicing. These findings not only provide fundamentally new insight into the mechanisms by which repeated exposure to cocaine regulates gene transcription in NAc, but they also provide important information concerning the basic mechanisms of transcriptional regulation in the brain *in vivo*.

Genome-wide mapping of histone modifications has emerged as a powerful means for characterizing the functional consequences of chromatin structure [15]. However, most available studies are derived from cultured cell systems during differentiation, development, or reprogramming. Whether similar rules defined in these homogeneous cell populations *in vitro* also apply to the brain *in vivo* is the key step to expand future epigenetic research. Our profiling of multiple histone marks in mouse NAc thus presents a much needed public reference resource for the neuro-epigenome, as well as detailed knowledge of global chromatin changes that occur in a discrete region of adult brain in response to repeated cocaine administration. We found that the basal patterns of the six histone marks studied are similar to those demonstrated in simpler systems. However, within these constraints, cocaine induced robust modifications in each of these marks at numerous genes and non-genic loci. We also found that the various histone marks carry different weights for transcriptional regulation, and that the combinatory pattern of modifications (chromatin signature) ultimately defines the transcriptional response. Our expectation is that analysis of still additional histone modifications will yield an ever more comprehensive and accurate epigenetic regulation network. Selective analysis of the cocaine-induced epigenomes of the several neuronal and non-neuronal cell types in NAc, something not yet technically feasible, would further improve our understanding of such networks. Nevertheless, our findings to date highlight the power of histone modification profiling for identifying diverse functional groups and target genes involved in cocaine action.

An unexpected finding of our study is the dominant contribution of changes in alternative splicing induced in NAc in response to chronic cocaine. In contrast to approximately 100 genes that show cocaine regulation of total transcript levels, we demonstrated an order of magnitude more genes that display altered splicing. These data indicate that previous studies that relied on microarray analysis and thereby focused on total gene transcription without discrimination of isoform differences dramatically underestimated the degree to which cocaine modifies the NAc transcriptome. Alternative pre-mRNA splicing is a major source of protein diversity in higher eukaryotes, a process particularly important for genes expressed in the brain [18,63,64]. Though there have been sporadic papers on splicing regulation of particular genes in addiction models [65-67], the present study is the first comprehensive analysis of splicing regulation in response to chronic cocaine. Given the fact that products of different splicing isoforms often serve unique cellular functions, the characterization of individual transcripts instead of the whole gene represents an important advance for understanding the molecular adaptations that underlie cocaine action.

Though alternative splicing was traditionally thought to be a post-transcriptional event, based largely on the primary sequence of the RNA, recent research has demonstrated that pre-mRNA splicing is intimately linked to transcription and the chromatin architecture of the gene [17]. The spliceosome is proposed to physically link to the transcriptional machinery through interactions between splicing factors and RNA pol II, and specific histone modifications have been shown to regulate alternative splicing in cell culture. For example, depolarization of cultured neurons triggers the skipping of exon 18 of the neural cell adhesion gene, a change accompanied by H3K9 hyper-acetylation around the exon [68]. The effect of depolarization can be further potentiated by treating the cells with a histone deacetylase inhibitor. As another example, the fibroblast growth factor receptor 2 (*Fgfr2*) gene is alternatively spliced into two isoforms, *Fgfr2-IIIb* and *-IIIc*[37]. The gene is enriched with H3K36me3 and H3K4me1 along the alternatively spliced region in mesenchymal cells where exon IIIc is transcribed, and with H3K27me3 and H3K4me3 in epithelial cells where exon IIIb is transcribed. Importantly, modulation of H3K36me3 or H3K4me3 levels by overexpression or down-regulation of their respective histone methyltransferases changes the tissue-specific alternative splicing pattern in a predictable fashion in cultured cells [37]. These observations suggest that localized changes in histone modification signatures along an alternatively spliced region can change splicing outcome. Furthermore, it provides a novel means of regulating gene transcription (splicing) through epigenetic manipulation.

However, studies to date have been mainly performed in cell culture with a candidate gene approach. How histone modifications relate to alternative splicing at a more global level, within the brain *in vivo* and in response to environmental stimuli, remains unknown. By obtaining genome-wide maps of several histone modifications within a discrete region of brain under chronic cocaine conditions coupled with genome-wide analysis of alternative splicing patterns, we have identified 29 chromatin signatures that differentially predict alterations in gene expression and, more specifically, regulation of alternative splicing. The genes are highly concentrated in certain functional groups. These findings indicate that control of pre-mRNA alternative splicing by histone modifications is a general feature of biological regulation. Moreover, an unbiased motif analysis inferred unique sets of transcription factors and splicing factors that are associated with individual chromatin signatures.

As a proof of principle, we selected to further analyze one candidate splicing factor, A2BP1, which has not previously been studied in cocaine action. A2BP1 is a neuron-specific splicing factor that promotes either exon inclusion or skipping. It has been implicated in several neurodevelopmental and neuropsychiatric disorders such as autism spectrum disorder, mental retardation, epilepsy, bipolar disorder, and schizophrenia [69]. The protein kinase WNK3 binds to A2BP1 and suppresses its splicing activity through a kinase activity-dependent cytoplasmic re-localization of A2BP1 [70]. Our observation of nuclear translocation of A2BP1 after repeated cocaine exposure suggests a robust role of A2BP1 in alternative splicing even though there is no change in total cellular levels of the protein. Increased nuclear levels of A2BP1 might facilitate adaptive alterations of pre-mRNA splicing of A2BP1 target transcripts that affect cocaine responses. Analysis of brain-specific *A2bp1* knockout mice revealed altered synaptic transmission, increased membrane excitability, and a predisposition to seizures [58]. Though few changes are seen in total transcript abundance, *A2bp1*-deficient brain displays a variety of splicing changes related to genes mediating synaptic transmission and membrane excitability. Similar implication of A2BP1 targets in neural transmission, neuronal development, and maturation genes has been demonstrated in autism spectrum disorder and human neural stem cell studies [61,69]. Through bioinformatic analysis, our genome-wide data predicted that A2BP1 associates with H3K4me3 in concert with the regulated splicing of target genes after repeated cocaine administration. Indeed, we verified that A2BP1 is associated with H3K4me3 in NAc in response to repeated cocaine administration. Moreover, we show that conditional knockdown of *A2bp1* from the adult NAc dramatically impairs rewarding responses to cocaine, and we confirmed regulation of several predicted A2BP1 target genes in NAc whose regulation by repeated cocaine is lost upon knockdown of this splicing factor. In the future, it will be interesting to further investigate the mechanisms by which cocaine triggers A2BP1 translocation to the nucleus and the means underlying A2BP1 regulation of its gene targets, work which will contribute to a better understanding of the molecular mechanism of cocaine action.

It is important to emphasize that sequencing data obtained from brain is inherently noisier than that obtained from simpler systems such as cultured cells. One prominent example is *Ttr*, which encodes transthyretin, important for thyroid hormone and retinol transport. It is highly enriched in choroid plexus [71], although expression in retina and certain central neurons has been reported [72,73]. As can be seen from Additional file 24, although our differential analysis shows that chronic cocaine regulates *Ttr* expression in NAc, this conclusion must be viewed with caution given the great variability in the cocaine and saline replicates. We therefore analyzed our entire differential gene list for genes that show similar large variance. Only three and two of the regulated genes show such variability in acute and chronic data, respectively, which underscores the importance of utilizing multiple statistical tests when evaluating RNA-seq datasets. The analyses also demonstrate that the differential lists reported in this study are generally sound, as substantiated further by the several levels of validation provided. Meanwhile, the source of the variability seen in *Ttr* and a small fraction of other genes remains unknown. One possible source of variability might be dissections of NAc. To gain insight into this possibility, we analyzed classes of genes known to be expressed either at very high levels or at relatively low levels in NAc versus surrounding brain regions, including the choroid plexus (Additional file 25). Among a list of over 100 choroid plexus-enriched genes [74] compared to striatum, only *Ttr* shows high variability; all of the others are consistently depleted in our datasets. The data also reveal strong consistency across replicates for NAc-enriched and -depleted genes. Thus, while dissecting a micronucleus from brain by necessity introduces some variability, these data argue for considerable consistency in our dissections. The analysis does, however, highlight systematic differences in expression levels of some genes seen across experiments: replicates are highly consistent within one experiment (for example, acute saline) but vary more between experiments (for example, acute versus chronic saline). Such 'batch' effects may reflect the different basal state of animals used at different times of experimentation, variability that is inherent in any *in vivo* experiment.

In any event, the results of this study confirm the important insight provided by the multiple platforms of analysis undertaken to better understand how repeated exposure to cocaine alters gene expression in NAc. By further mining these data, and carrying out similar analyses at different time points of cocaine exposure and cocaine treatment paradigms with additional epigenetic marks, it will be possible to ultimately explore the complete complex program of gene regulation that underlies important aspects of drug addiction.

## Materials and methods

### Cocaine treatment and nucleus accumbens dissection

Adult male C57BL/6 J mice (Jackson) 8 weeks old were used in this study. They were housed five per cage on a 12-h light-dark cycle at constant temperature (23°C) with free access to food and water *ad libitum*. Animals were habituated for at least 1 week before experimentation. For repeated cocaine treatment, animals received daily intraperitoneal injections for seven consecutive days of cocaine (Sigma-Aldrich, St. Louis, MO, USA) at 20 mg/kg body weight ('repeated cocaine'). Mice were used 24 h after the final injection. For acute cocaine treatment, mice received only one injection of cocaine at 20 mg/kg body weight on day seven after six daily intraperitoneal saline injections. Control mice for all groups received daily saline injections for seven days. Bilateral 14-gauge NAc punches were taken from each animal 24 h after the last injection. All animal protocols were approved by the Institutional Animal Care and Use Committee of Mount Sinai.

### Locomotor activity assay

Mouse locomotor activity was tested as previously described [8]. In brief, mice were injected with saline or cocaine (20 mg/kg) at the same time each day and placed in standard rat cages located inside a Photobeam Activity System (San Diego Instruments, San Diego, CA, USA). On day 0, mice were habituated to the cage for 30 minutes and then given a saline injection. On days 1 to 7, mice were given injections of cocaine. Horizontal ambulation was measured for 30 minutes after all injections.

### RNA-seq

Brain samples were homogenized in Trizol and processed according to the manufacturer’s instructions. RNA was purified with RNeasy Micro columns and Bioanalyzer confirmed that the RNA integrity numbers were >8.0. Total RNA (4 μg) was used for mRNA library construction following instructions of Illumina mRNA sample prep kit (catalog number RS-100-0801). Please refer to Additional file 7 for details. The RNA-seq read alignment and differential analysis were done using TopHat [22] and Cufflinks [24] packages. For our initial analysis, cutoffs were set as FDR <10%, fold change >1.25, and RPKM >1 for treatment and control groups. For subsequent broader analyses, we used an FDR cutoff of only <10%.

### ChIP-seq

ChIP was performed as previously described [9,13]. Antibodies were all ChIP grade from Abcam, Cambridge, MA, USA. Around 10 nanograms of input DNA or pull-down DNA were used for sequencing library preparation following the instructions of Illumina’s ChIP-seq sample prep kit (catalog number IP-102-1001). Please refer to Additional file 7 for details. The ChIP-seq read alignment was done using Illumina’s CASAVA pipeline. Please refer to Additional file 7 for details on further filtering. Differential analysis was done by diffReps [32] with window size 200 bp and moving size 20 bp. A FDR <10% was used as the significance cutoff. Global visualization for the ChIP-seq data was accomplished with a program called ngs.plot [30] (Additional file 7). Basal level peak calling was carried out using MACS [75] with the three saline replicates pooled and DNA input samples used as background.

### Chromatin signatures

The reference gene database was analyzed to extract the genomic coordinates for the six types of exons and neighboring intronic regions. These genomic coordinates were then compared against the ChIP-seq alignment files to determine the fold changes that were further assembled into chromatin signatures for clustering (see Additional file 7 for more details).

### Nuclear protein isolation, co-immunoprecipitation, and western blotting

Nuclear protein isolation was done following a published protocol [76]. Please refer to Additional file 7 for details. Immunoprecipitation was performed following a standard protocol with H3K4me3 antibody from Millipore, Billerica, MA, USA. Either nuclear protein or immunoprecipitated proteins were used for western blotting as described previously [76]. Antibodies used in this experiment were A2BP1 (1:500; Abcam) and histone 3 (1:1,000; Abcam).

### Nanostring assay

High quality RNA (RNA integrity number >8) was selected based on bioanalyzer examination. Up to 1,000 ng of total RNA samples were submitted to NanoString for analysis with the Gene Expression Assay. The code set was designed by the company with unique sequences. Raw counts for each assay were collected and normalized with the NanoString data analysis software nSolver. Both positive control and reference housekeeping genes were utilized for normalization of read counts.

### *A2bp1* knockout mice, stereotaxic surgery, and conditional place preference

Adult (6 to 8 weeks old) *A2bp1*^*loxP/*loxP^ mice were purchased from Jackson (stock number 014089) [58]. AAV-Cre and AAV-GFP vectors were used, and stereotaxic intra-NAc injections were performed, as reported [77]. Viral injection sites were verified by confirming the GFP signal in NAc slices under the microscope. Viral knockdown of *A2bp1* was confirmed using quantitative PCR.

A standard, unbiased CPP procedure was utilized as described [77]. In brief, 3 to 4 weeks after viral injection, when AAV-mediated expression is maximal, animals were pretested for 20 minutes in a photo-beam monitored box with free access to environmentally distinct chambers. The mice were then arranged into control and experimental groups with balanced pretest scores. Then mice underwent four 30-minute training sessions (saline in the morning and cocaine in the afternoon) over two days. On the test day, mice had 20 minutes of unrestricted access to all chambers and a CPP score was assigned by subtracting the time spent in the cocaine-paired chamber from the time spent in the saline-paired chamber. Cocaine was injected intraperitoneally at 7.5 mg/kg.

### Data access

All the ChIP-seq and RNA-seq data have been deposited into the Gene Expression Omnibus with accession number GSE42811 with the exception of cocaine replicates 1 and 2 and saline replicates 1, 2, and 3 of the H3K9me3 ChIP-seq, which were previously deposited in the Gene Expression Omnibus, submission GSE24850.

## Abbreviations

AAV: adeno-associated virus; bp: base pair; ChIP: chromatin immunoprecipitation; CPP: conditioned place preference; GFP: green fluorescent protein; GO: gene ontology; NAc: nucleus accumbens; PCR: polymerase chain reaction; RNA polII: RNA polymerase II; TSS: transcriptional start site.

## Competing interests

The authors declare that they have no competing interests.

## Authors’ contributions

EJN, LS, and JF conceived and designed the experiments. LS provided analytic tools and analyzed the data. XL, IP, NS, JF, and EJN participated in ChIP-seq and RNA-seq data analysis. JF and MW generated the RNA-seq and ChIP-seq data. JF performed the nanostring, quantitative PCR, and quantitative ChIP experiments. DF, JF, and MC performed nuclear protein isolation, co-immunoprecipitation and western blotting. JF, VV, and JK carried out stereotaxic surgery and CPP experiments. QL contributed locomotor data. DF, VV, IM, PK, CD, BL, VS, and QL participated in generating various datasets. LS, JF, and EJN wrote the manuscript. All authors read and commented on the manuscript. All authors read and approved the final manuscript.

## Supplementary Material

Additional file 1: Figure S1 Locomotor sensitization to repeated cocaine. Mice received daily cocaine (20 mg/kg) or saline injections for 7 days and were evaluated for locomotor activity on days 1, 3, and 7. A significant (two-way ANOVA) main effect of day (F2,36 = 4.47, *P* < 0.01) and drug (F1,36 = 36.37, *P* < 0.0001) and an interaction between day and drug (F2,36 = 7.38, *P* = 0.002) was observed (cocaine, N = 8; saline, N = 12). Bonferroni post hoc analysis reveals significant increases in locomotor activity after cocaine versus saline on days 1 and 3 (*P < 0.01) and significantly greater activity on day 7 versus day 1 (**P < 0.01). Data are presented as mean ± standard error of the mean. Click here for file

Additional file 2: Table S1 RNA-seq quality control metrics for acute and chronic data. Click here for file

Additional file 3: Table S2 Differential RNA-seq lists. Differential gene lists from repeated and acute cocaine RNA-seq experiments; differential splicing lists from repeated cocaine RNA-seq experiments. Cuffdiff was used to perform differential analysis for various transcriptomic events. Click here for file

Additional file 4: Figure S2 Sample splicing screenshots. Genome browser screenshots of alternative splicing examples from chronic cocaine RNA-seq experiments. The red and green tracks represent normalized RNA-seq coverage in cocaine and saline. The data scale is the same for both cocaine and saline. The schemes of an alternatively spliced transcript and a contrast transcript are shown at the bottom. The black boxes highlight the alternative regions that show different expression changes from the rest of the gene body. The asterisk indicates that the isoform is predicted to be significantly changed. FC, fold change. **(A)***Ttc23*: ENSMUST00000107470 (or TCONS_00070790), log2 FC = 3.7, q-value = 2E-4; ENSMUST00000126093 (or TCONS_00070785), log2 FC = 0.7, q-value = 0.7. **(B)***Sp100*: ENSMUST00000147552 (or TCONS_00001011), log2 FC = 2.8, q-value = 0.01; ENSMUST00000153574 (or TCONS_00001012), log2 FC = 1.3, q-value = 0.7. **(C)***Sept7*: ENSMUST00000115272 (or TCONS_00079740), log2 FC = -0.3, q-value = 0.007; ENSMUST00000060080 (or TCONS_00079741), log2 FC = 0.3, q-value = 1. Click here for file

Additional file 5: Figure S3 RNAseq nanostring validation. Nanostring validation of cocaine-induced changes in RNA expression in NAc. A separate cohort of animals was used to validate RNA-seq results. Normalized Nanostring read counts are shown on the y-axis. All genes display the same direction of change with significance as seen with RNA-seq. Error bars are mean ± standard error of the mean derived from 14 cocaine treated and 14 saline treated samples. *P < 0.05, **P < 0.01. Click here for file

Additional file 6: Table S3 GO term enrichment of genes that have altered splicing. The altered splicing group combines the genes that contain alternative promoter usage and/or alternative splicing. DAVID is used to perform GO analysis. Only three GO categories are used: biological process, cellular component, and molecular function. Click here for file

Additional file 7 Extended experimental procedures. Click here for file

Additional file 8: Table S4 ChIP-seq sample read statistics. #Uniq = number of uniquely aligned reads; #Rmdup = number of reads after removing duplicates (Additional file 7); #TotRead = total number of reads combining three replicates; #TotNuc = total number of nucleotides. Click here for file

Additional file 9: Figure S4 Global enrichment plots and numbers of differential events. Each panel includes five sub-figures for the enrichment, using data pooled from the three biological replicates, of an epigenomic mark at TSSs, gene bodies, transcriptional end sites, and cocaine up-regulated sites and down-regulated sites. Y-axes represent the normalized coverage (RPM) that is averaged across all genomic regions. **(A)** H3K4me1. **(B)** H3K4me3. **(C)** H3K9me2. **(D)** H3K9me3. **(E)** H3K27me3. **(F)** H3K36me3. **(G)** RNA pol II. **(H)** Number of differential events for the seven epigenomic marks. Click here for file

Additional file 10: Table S5 Differential sites for the seven epigenomic marks. diffReps is used to identify differential sites for each of the seven epigenomic marks. A FDR cutoff of <10% was used to choose the sites that are significant. Click here for file

Additional file 11: Figure S5 Distribution of basal peaks for the seven epigenomic marks. **(A)** H3K4me1. **(B)** H3K4me3. **(C)** H3K9me2. **(D)** H3K9me3. **(E)** H3K27me3. **(F)** H3K36me3. **(G)** RNA pol II. Click here for file

Additional file 12: Figure S6 Heatmap showing the enrichment of the top 30 and custom pathways among the seven marks. The darkness of each grid represents the statistical significance of enrichment. Click here for file

Additional file 13: Table S6 Enrichment analysis of ChIP-seq differential sites. After the differential sites are mapped to promoter or gene body, the genes that contain the differential sites are uploaded to IPA for enrichment analysis. The enriched canonical pathways or customized gene lists are extracted. Each value represents -log10(*P*-value) of enrichment. Co-occurrence score (Additional file 7) is used to rank the pathways in descending order. Click here for file

Additional file 14: Figure S7 Differential sites to exon center distance density plots (related to Figure 3). The distance between each differential site and the closest exon center was calculated. The exons were further classified into three categories: promoter, internal, and polyA. The density for the distance within a 10 kb window of the exon center of each type was calculated. Each panel represents an epigenomic mark. **(A)** H3K4me1. **(B)** H3K4me3. **(C)** H3K9me2. **(D)** H3K9me3. **(E)** H3K27me3. **(F)** H3K36me3. **(G)** RNA pol II. Click here for file

Additional file 15: Figure S8 Coverage plots for the seven marks at six different types of exons (see main text). The exons are further classified based on the RNA-seq RPKM of the corresponding transcript: 'High' (≥10), 'Medium' (≥1 and <10), and 'Low' (<1). **(A)** H3K4me1. **(B)** H3K4me3. **(C)** H3K9me2. **(D)** H3K9me3. **(E)** H3K27me3. **(F)** H3K36me3. **(G)** RNA pol II. Click here for file

Additional file 16: Figure S9 Construction of cocaine-induced chromatin signatures. All chromatin signatures are put into a signature matrix with each row being a transcript and each column being the log fold change of each mark at each genomic region. K-means clustering was performed on the signature matrix to group transcripts into signature clusters that share common chromatin modification patterns. The regions that show significant chromatin changes were extracted to perform motif analysis to identify potential splicing and transcription factors. Click here for file

Additional file 17: Table S7 Genome-wide association between chromatin modification and transcriptional change. Based on the chromatin modification at each genomic region, transcripts are separated into up-regulated, down- regulated, and non-significant (Additional file 7). The chromatin-up and -down transcripts are correlated with transcripts that show expression change using Fisher’s exact test. This generates four combinations ('s' = chromatin modification, 'e' = expression change): s.up.e.up; s.up.e.down; s.down.e.up; s.down.e.down. The *P*-values were adjusted using the BH [78] method and a FDR cutoff of <10% was used to select mark-region combinations. The analysis was first done with the enhancer regions included and then repeated with the enhancers removed. Click here for file

Additional file 18: Figure S11 Chromatin modification heatmap for 29 signature clusters. A merged heatmap for all 29 signature clusters with transcripts as rows and mark-region combinations as columns. The color key indicates log2 fold changes. Different clusters are labeled by different colors. Click here for file

Additional file 19: Table S8 Enriched functional terms and canonical pathways among the 29 signature clusters. IPA was used to find the enriched biological functions and canonical pathways among the signature clusters. The co-occurrence score was then used to rank the enriched terms in descending order. Click here for file

Additional file 20 **Motif intermediate results.** This zip file contains the textual outputs from motif analysis. The motifs found by MEME [42] were first combined using the Bayesian motif clustering [79] method and then matched with known motifs. Further explanations are provided in the enclosed README file. Click here for file

Additional file 21: Figure S10 Quantitative ChIP validation of cocaine-induced changes in H3K4me3 in NAc. A separate cohort of animals was used to validate ChIP-seq data. All genomic sites tested display the same direction of change with significance as seen with ChIP-seq. Error bars are mean ± standard error of the mean derived from 8 to 14 replicates per condition. *P < 0.05, **P < 0.01. Click here for file

Additional file 22: Table S9 A2BP1 and H3K4me3 overlapping genes that are also cocaine-regulated. Click here for file

Additional file 23: Table S10 A2BP1 H3K4me3 cocaine-regulated gene functional enrichment. Functional enrichment for the genes in Additional file 22. IPA was used to identify enriched biological functions for the genes listed in Additional file 22 restricted to the central nervous system. Click here for file

Additional file 24: Table S11 Inter-replicate variability of differential genes. Variability was measured by the coefficient of variance (CV), which equals the mean divided by the standard deviation. The mean and CV values for both cocaine and saline conditions are shown. Click here for file

Additional file 25: Table S12 Quality control of RNA-seq data. RPKM values are shown for the three saline samples from the acute experiment and from the chronic experiment. MSN-enriched genes: genes known from previous studies to be enriched in striatal (including NAc) medium spiny neurons. MSN-depleted genes: genes encoding related neurotransmitter and neuropeptide system products known from previous studies to be expressed at low levels in NAc. Choroid plexus-enriched genes: genes known from previous studies to be enriched in choroid plexus, although many of these genes are also known to be expressed in neurons. Click here for file
