# Erratum to: Chronic cocaine-regulated epigenomic changes in mouse nucleus accumbens

**DOI:** 10.1186/s13059-015-0789-8

**Published:** 2015-10-14

**Authors:** Jian Feng, Matthew Wilkinson, Xiaochuan Liu, Immanuel Purushothaman, Deveroux Ferguson, Vincent Vialou, Ian Maze, Ningyi Shao, Pamela Kennedy, JaWook Koo, Caroline Dias, Benjamin Laitman, Victoria Stockman, Quincey LaPlant, Michael E Cahill, Eric J Nestler, Li Shen

**Affiliations:** Fishberg Department of Neuroscience and Friedman Brain Institute, Icahn School of Medicine at Mount Sinai, One Gustave L. Levy Place, Box 1065, New York, NY 10029 USA

During the typesetting of the final version of the article [1] Tables S2, S8 and S11 were duplicated and Tables S1, S7 and S9 are missing. We apologize for the mistake that led to the loss of some of the additional files.

In the final version of the article:

Additional file 2: Table S1 is wrongly replaced as Table S2.

Additional file 17: Table S7 is wrongly replaced as Table S8.

Additional file 22: Table S9 is wrongly replaced as Table S11

Please see missing Additional files 2, 17 and 22 below.

All Additional files were correct in the provisional version of the article. Below please find the correct full list of Additional Files associated with this article.

**Additional file 1: Figure S1**. Locomotor sensitization to repeated cocaine. Mice received daily cocaine (20 mg/kg) or saline injections for 7 days and were evaluated for locomotor activity on days 1, 3, and 7. A significant (two-way ANOVA) main effect of day (F2,36 = 4.47, *P* < 0.01) and drug (F1,36 = 36.37, *P* < 0.0001) and an interaction between day and drug (F2,36 = 7.38, *P* = 0.002) was observed (cocaine, N = 8; saline, N = 12). Bonferroni post hoc analysis reveals significant increases in locomotor activity after cocaine versus saline on days 1 and 3 (*P < 0.01) and significantly greater activity on day 7 versus day 1 (**P < 0.01). Data are presented as mean ± standard error of the mean.

**Additional file 2: Table S1**. RNA-seq quality control metrics for acute and chronic data.

**Additional file 3: Table S2.** Differential RNA-seq lists. Differential gene lists from repeated and acute cocaine RNA-seq experiments; differential splicing lists from repeated cocaine RNA-seq experiments. Cuffdiff was used to perform differential analysis for various transcriptomic events.

**Additional file 4: Figure S2.** Sample splicing screenshots. Genome browser screenshots of alternative splicing examples from chronic cocaine RNA-seq experiments. The red and green tracks represent normalized RNA-seq coverage in cocaine and saline. The data scale is the same for both cocaine and saline. The schemes of an alternatively spliced transcript and a contrast transcript are shown at the bottom. The black boxes highlight the alternative regions that show different expression changes from the rest of the gene body. The asterisk indicates that the isoform is predicted to be significantly changed. FC, fold change. **(A)***Ttc23*: ENSMUST00000107470 (or TCONS_00070790), log2 FC = 3.7, q-value = 2E-4; ENSMUST00000126093 (or TCONS_00070785), log2 FC = 0.7, q-value = 0.7. **(B)***Sp100*: ENSMUST00000147552 (or TCONS_00001011), log2 FC = 2.8, q-value = 0.01; ENSMUST00000153574 (or TCONS_00001012), log2 FC = 1.3, q-value = 0.7. **(C)***Sept7*: ENSMUST00000115272 (or TCONS_00079740), log2 FC = −0.3, q-value = 0.007; ENSMUST00000060080 (or TCONS_00079741), log2 FC = 0.3, q-value = 1.

**Additional file 5: Figure S3**. RNAseq nanostring validation. Nanostring validation of cocaine-induced changes in RNA expression in NAc. A separate cohort of animals was used to validate RNA-seq results. Normalized Nanostring read counts are shown on the y-axis. All genes display the same direction of change with significance as seen with RNA-seq. Error bars are mean ± standard error of the mean derived from 14 cocaine treated and 14 saline treated samples. *P < 0.05, **P < 0.01.

**Additional file 6: Table S3.** GO term enrichment of genes that have altered splicing. The altered splicing group combines the genes that contain alternative promoter usage and/or alternative splicing. DAVID is used to perform GO analysis. Only three GO categories are used: biological process, cellular component, and molecular function.

**Additional file 7: Extended experimental procedures.**

**Additional file 8: Table S4.** ChIP-seq sample read statistics. #Uniq = number of uniquely aligned reads; #Rmdup = number of reads after removing duplicates (Additional file 7); #TotRead = total number of reads combining three replicates; #TotNuc = total number of nucleotides.

**Additional file 9: Figure S4.** Global enrichment plots and numbers of differential events. Each panel includes five sub-figures for the enrichment, using data pooled from the three biological replicates, of an epigenomic mark at TSSs, gene bodies, transcriptional end sites, and cocaine up-regulated sites and down-regulated sites. Y-axes represent the normalized coverage (RPM) that is averaged across all genomic regions. **(A)** H3K4me1. **(B)** H3K4me3. **(C)** H3K9me2. **(D)** H3K9me3. **(E)** H3K27me3. **(F)** H3K36me3. **(G)** RNA pol II. **(H)** Number of differential events for the seven epigenomic marks.

**Additional file 10: Table S5.** Differential sites for the seven epigenomic marks. diffReps is used to identify differential sites for each of the seven epigenomic marks. A FDR cutoff of <10 % was used to choose the sites that are significant.

**Additional file 11: Figure S5.** Distribution of basal peaks for the seven epigenomic marks. **(A)** H3K4me1. **(B)** H3K4me3. **(C)** H3K9me2. **(D)** H3K9me3. **(E)** H3K27me3. **(F)** H3K36me3. **(G)** RNA pol II.

**Additional file 12: Figure S6.** Heatmap showing the enrichment of the top 30 and custom pathways among the seven marks. The darkness of each grid represents the statistical significance of enrichment.

**Additional file 13: Table S6.** Enrichment analysis of ChIP-seq differential sites. After the differential sites are mapped to promoter or gene body, the genes that contain the differential sites are uploaded to IPA for enrichment analysis. The enriched canonical pathways or customized gene lists are extracted. Each value represents -log10(*P*-value) of enrichment. Co-occurrence score (Additional file 7) is used to rank the pathways in descending order.

**Additional file 14: Figure S7**. Differential sites to exon center distance density plots (related to Figure 3). The distance between each differential site and the closest exon center was calculated. The exons were further classified into three categories: promoter, internal, and polyA. The density for the distance within a 10 kb window of the exon center of each type was calculated. Each panel represents an epigenomic mark. **(A)** H3K4me1. **(B)** H3K4me3. **(C)** H3K9me2. **(D)** H3K9me3. **(E)** H3K27me3. **(F)** H3K36me3. **(G)** RNA pol II.

**Additional file 15: Figure S8.** Coverage plots for the seven marks at six different types of exons (see main text). The exons are further classified based on the RNA-seq RPKM of the corresponding transcript: ‘High’ (≥10), ‘Medium’ (≥1 and <10), and ‘Low’ (<1). **(A)** H3K4me1. **(B)** H3K4me3. **(C)** H3K9me2. **(D)** H3K9me3. **(E)** H3K27me3. **(F)** H3K36me3. **(G)** RNA pol II.

**Additional file 16: Figure S9.** Construction of cocaine-induced chromatin signatures. All chromatin signatures are put into a signature matrix with each row being a transcript and each column being the log fold change of each mark at each genomic region. K-means clustering was performed on the signature matrix to group transcripts into signature clusters that share common chromatin modification patterns. The regions that show significant chromatin changes were extracted to perform motif analysis to identify potential splicing and transcription factors.

**Additional file 17: Table S7.** Genome-wide association between chromatin modification and transcriptional change. Based on the chromatin modification at each genomic region, transcripts are separated into up-regulated, down- regulated, and non-significant (Additional file 7). The chromatin-up and -down transcripts are correlated with transcripts that show expression change using Fisher’s exact test. This generates four combinations (‘s’ = chromatin modification, ‘e’ = expression change): s.up.e.up; s.up.e.down; s.down.e.up; s.down.e.down. The *P*-values were adjusted using the BH [78] method and a FDR cutoff of <10 % was used to select mark-region combinations. The analysis was first done with the enhancer regions included and then repeated with the enhancers removed.

**Additional file 18: Figure S11.** Chromatin modification heatmap for 29 signature clusters. A merged heatmap for all 29 signature clusters with transcripts as rows and mark-region combinations as columns. The color key indicates log2 fold changes. Different clusters are labeled by different colors.

**Additional file 19: Table S8.** Enriched functional terms and canonical pathways among the 29 signature clusters. IPA was used to find the enriched biological functions and canonical pathways among the signature clusters. The co-occurrence score was then used to rank the enriched terms in descending order.

**Additional file 20: Motif intermediate results.** This zip file contains the textual outputs from motif analysis. The motifs found by MEME [42] were first combined using the Bayesian motif clustering [79] method and then matched with known motifs. Further explanations are provided in the enclosed README file.

**Additional file 21: Figure S10.** Quantitative ChIP validation of cocaine-induced changes in H3K4me3 in NAc. A separate cohort of animals was used to validate ChIP-seq data. All genomic sites tested display the same direction of change with significance as seen with ChIP-seq. Error bars are mean ± standard error of the mean derived from 8 to 14 replicates per condition. *P < 0.05, **P < 0.01.

**Additional file 22: Table S9.** A2BP1 and H3K4me3 overlapping genes that are also cocaine-regulated.

**Additional file 23: Table S10.** A2BP1 H3K4me3 cocaine-regulated gene functional enrichment. Functional enrichment for the genes in Additional file 22. IPA was used to identify enriched biological functions for the genes listed in Additional file 22 restricted to the central nervous system.

**Additional file 24: Table S11.** Inter-replicate variability of differential genes. Variability was measured by the coefficient of variance (CV), which equals the mean divided by the standard deviation. The mean and CV values for both cocaine and saline conditions are shown.

**Additional file 25: Table S12.** Quality control of RNA-seq data. RPKM values are shown for the three saline samples from the acute experiment and from the chronic experiment. MSN-enriched genes: genes known from previous studies to be enriched in striatal (including NAc) medium spiny neurons. MSN-depleted genes: genes encoding related neurotransmitter and neuropeptide system products known from previous studies to be expressed at low levels in NAc. Choroid plexus-enriched genes: genes known from previous studies to be enriched in choroid plexus, although many of these genes are also known to be expressed in neurons.

## Additional files

Additional file 2:
**Table S1.** RNA-seq quality control metrics for acute and chronic data. (XLSX 13 kb) Additional file 17:
**Table S7.** Genome-wide association between chromatin modification and transcriptional change. Based on the chromatin modification at each genomic region, transcripts are separated into up-regulated, down- regulated, and non-significant (Additional file 7). The chromatin-up and -down transcripts are correlated with transcripts that show expression change using Fisher’s exact test. This generates four combinations ('s' = chromatin modification, 'e' = expression change): s.up.e.up; s.up.e.down; s.down.e.up; s.down.e.down. The P-values were adjusted using the BH [78] method and a FDR cutoff of <10% was used to select mark-region combinations. The analysis was first done with the enhancer regions included and then repeated with the enhancers removed. (XLSX 18 kb) Additional file 22:
**Table S9.** A2BP1 and H3K4me3 overlapping genes that are also cocaine-regulated. (XLSX 28 kb)
