# Supplementary figures and images for: Chronic cocaine-regulated epigenomic changes in mouse nucleus accumbens
Source: Genome Biol. 2014 Apr 22;15(4):R65. doi: 10.1186/gb-2014-15-4-r65 (PMC4073058; doi:10.1186/gb-2014-15-4-r65)

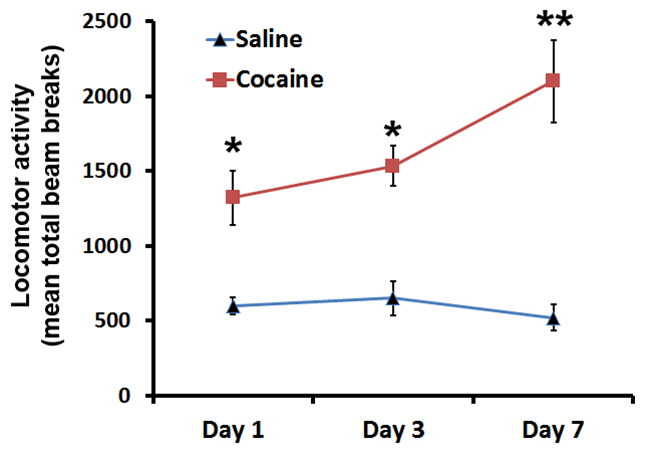

Supplement: Additional file 1: Figure S1 — Locomotor sensitization to repeated cocaine. Mice received daily cocaine (20 mg/kg) or saline injections for 7 days and were evaluated for locomotor activity on days 1, 3, and 7. A significant (two-way ANOVA) main effect of day (F2,36 = 4.47, P < 0.01) and drug (F1,36 = 36.37, P < 0.0001) and an interaction between day and drug (F2,36 = 7.38, P = 0.002) was observed (cocaine, N = 8; saline, N = 12). Bonferroni post hoc analysis reveals significant increases in locomotor activity after cocaine versus saline on days 1 and 3 (*P < 0.01) and significantly greater activity on day 7 versus day 1 (**P < 0.01). Data are presented as mean ± standard error of the mean. [file gb-2014-15-4-r65-S1.tiff]

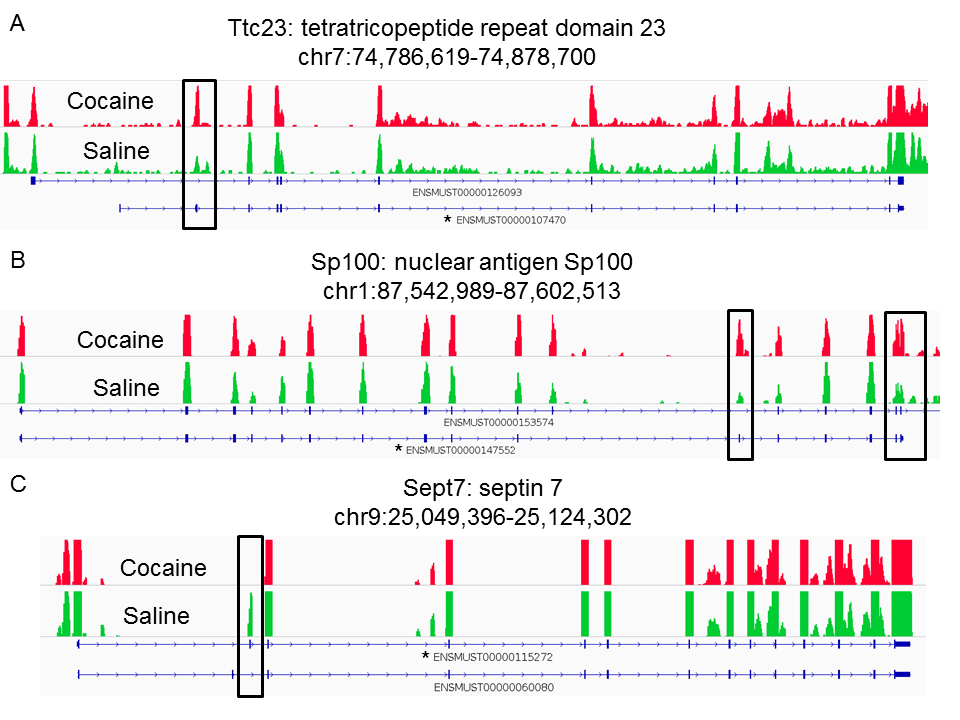

Supplement: Additional file 4: Figure S2 — Sample splicing screenshots. Genome browser screenshots of alternative splicing examples from chronic cocaine RNA-seq experiments. The red and green tracks represent normalized RNA-seq coverage in cocaine and saline. The data scale is the same for both cocaine and saline. The schemes of an alternatively spliced transcript and a contrast transcript are shown at the bottom. The black boxes highlight the alternative regions that show different expression changes from the rest of the gene body. The asterisk indicates that the isoform is predicted to be significantly changed. FC, fold change. (A)Ttc23: ENSMUST00000107470 (or TCONS_00070790), log2 FC = 3.7, q-value = 2E-4; ENSMUST00000126093 (or TCONS_00070785), log2 FC = 0.7, q-value = 0.7. (B)Sp100: ENSMUST00000147552 (or TCONS_00001011), log2 FC = 2.8, q-value = 0.01; ENSMUST00000153574 (or TCONS_00001012), log2 FC = 1.3, q-value = 0.7. (C)Sept7: ENSMUST00000115272 (or TCONS_00079740), log2 FC = -0.3, q-value = 0.007; ENSMUST00000060080 (or TCONS_00079741), log2 FC = 0.3, q-value = 1. [file gb-2014-15-4-r65-S4.png]

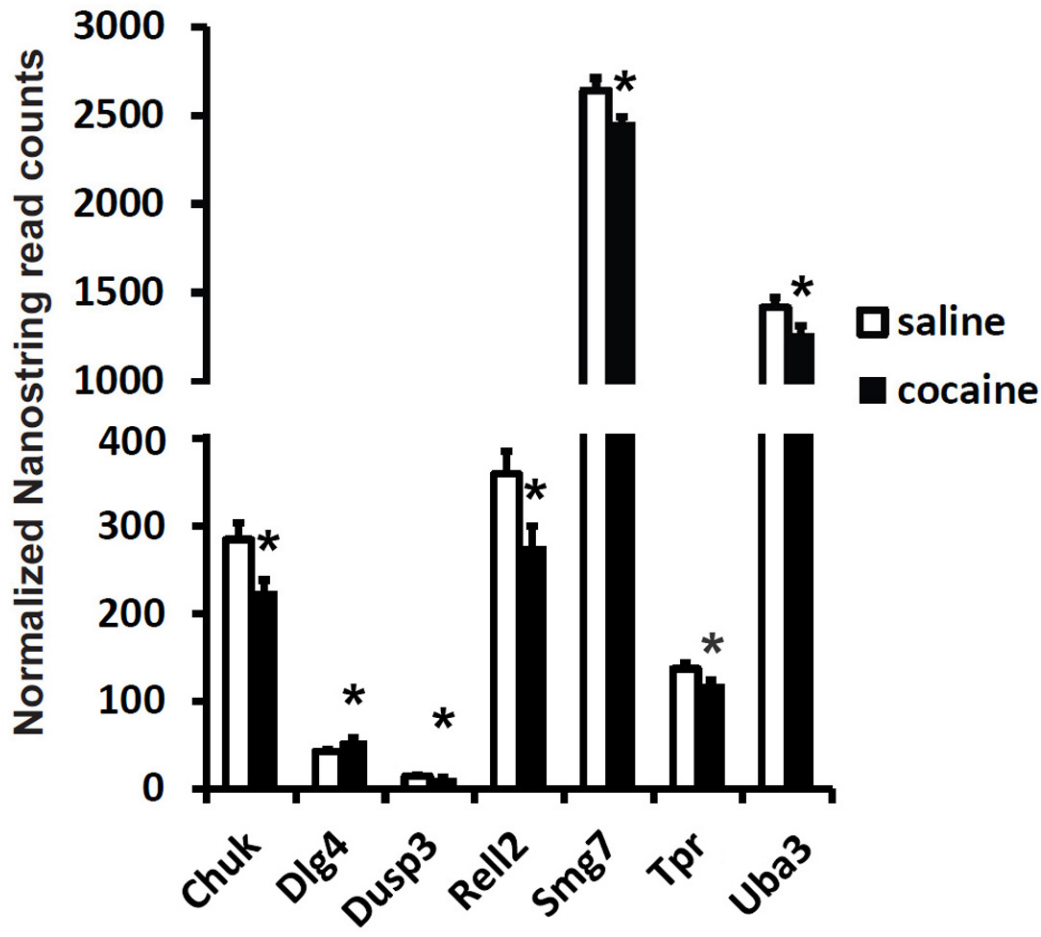

Supplement: Additional file 5: Figure S3 — RNAseq nanostring validation. Nanostring validation of cocaine-induced changes in RNA expression in NAc. A separate cohort of animals was used to validate RNA-seq results. Normalized Nanostring read counts are shown on the y-axis. All genes display the same direction of change with significance as seen with RNA-seq. Error bars are mean ± standard error of the mean derived from 14 cocaine treated and 14 saline treated samples. *P < 0.05, **P < 0.01. [file gb-2014-15-4-r65-S5.pdf]

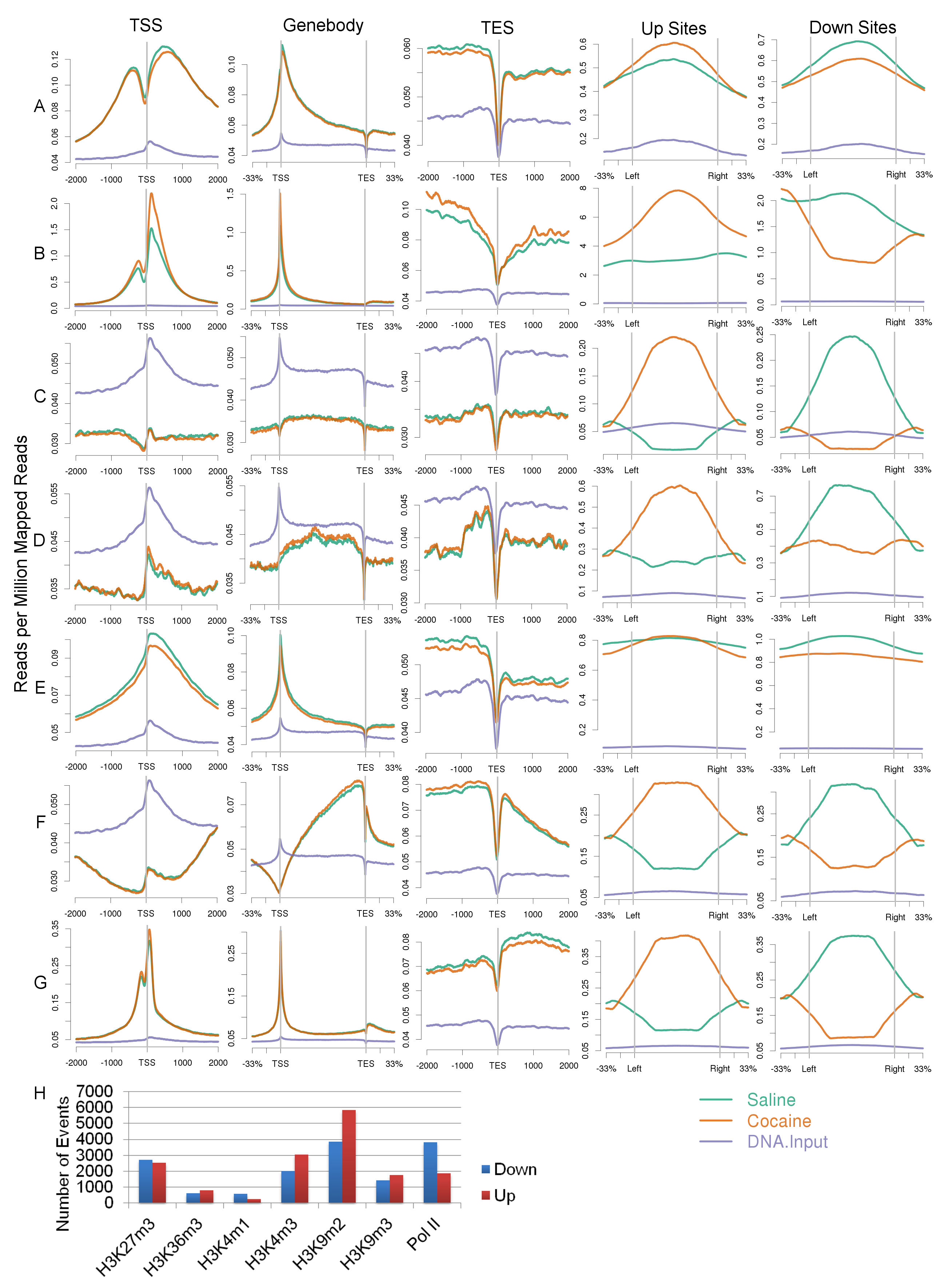

Supplement: Additional file 9: Figure S4 — Global enrichment plots and numbers of differential events. Each panel includes five sub-figures for the enrichment, using data pooled from the three biological replicates, of an epigenomic mark at TSSs, gene bodies, transcriptional end sites, and cocaine up-regulated sites and down-regulated sites. Y-axes represent the normalized coverage (RPM) that is averaged across all genomic regions. (A) H3K4me1. (B) H3K4me3. (C) H3K9me2. (D) H3K9me3. (E) H3K27me3. (F) H3K36me3. (G) RNA pol II. (H) Number of differential events for the seven epigenomic marks. [file gb-2014-15-4-r65-S9.tiff]

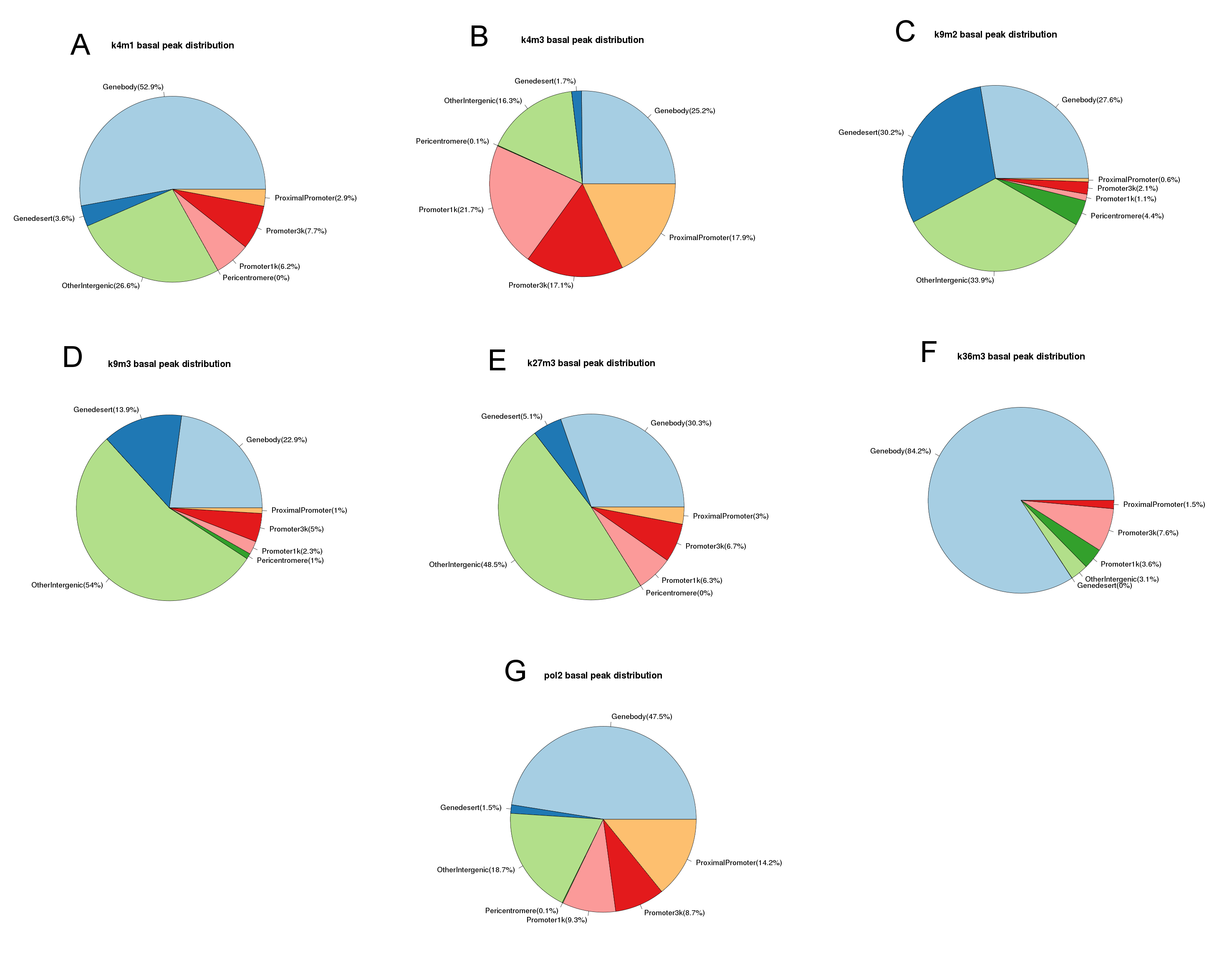

Supplement: Additional file 11: Figure S5 — Distribution of basal peaks for the seven epigenomic marks. (A) H3K4me1. (B) H3K4me3. (C) H3K9me2. (D) H3K9me3. (E) H3K27me3. (F) H3K36me3. (G) RNA pol II. [file gb-2014-15-4-r65-S11.tiff]

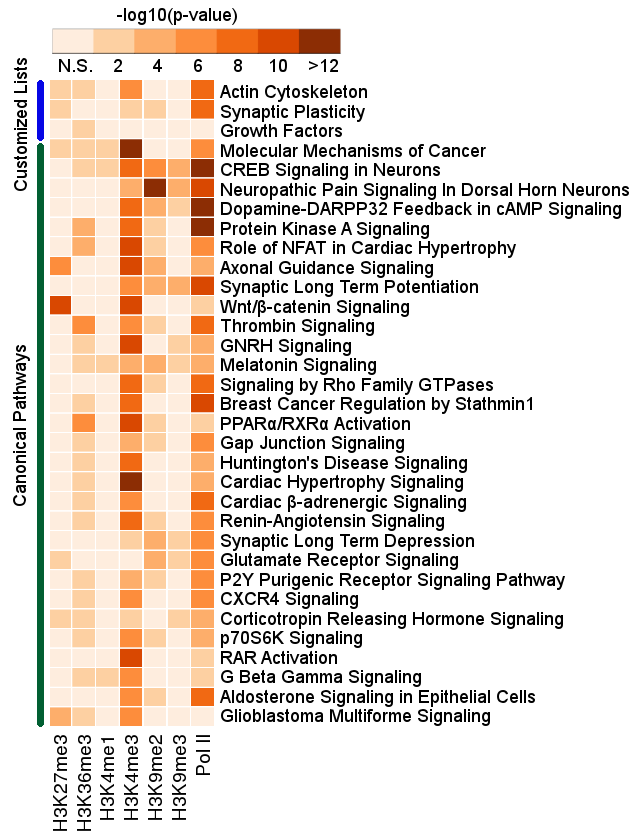

Supplement: Additional file 12: Figure S6 — Heatmap showing the enrichment of the top 30 and custom pathways among the seven marks. The darkness of each grid represents the statistical significance of enrichment. [file gb-2014-15-4-r65-S12.tiff]

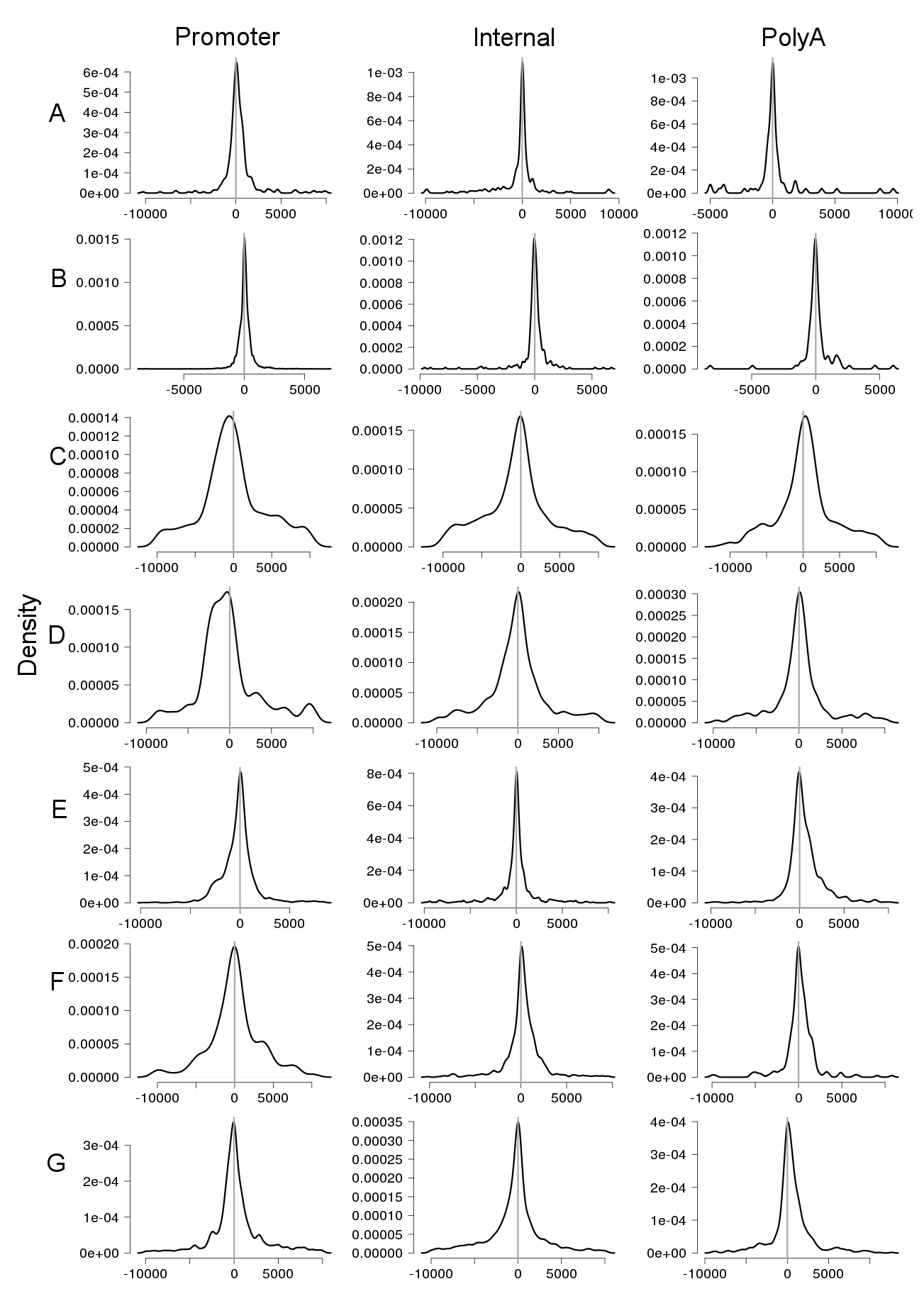

Supplement: Additional file 14: Figure S7 — Differential sites to exon center distance density plots (related to Figure 3). The distance between each differential site and the closest exon center was calculated. The exons were further classified into three categories: promoter, internal, and polyA. The density for the distance within a 10 kb window of the exon center of each type was calculated. Each panel represents an epigenomic mark. (A) H3K4me1. (B) H3K4me3. (C) H3K9me2. (D) H3K9me3. (E) H3K27me3. (F) H3K36me3. (G) RNA pol II. [file gb-2014-15-4-r65-S14.tiff]

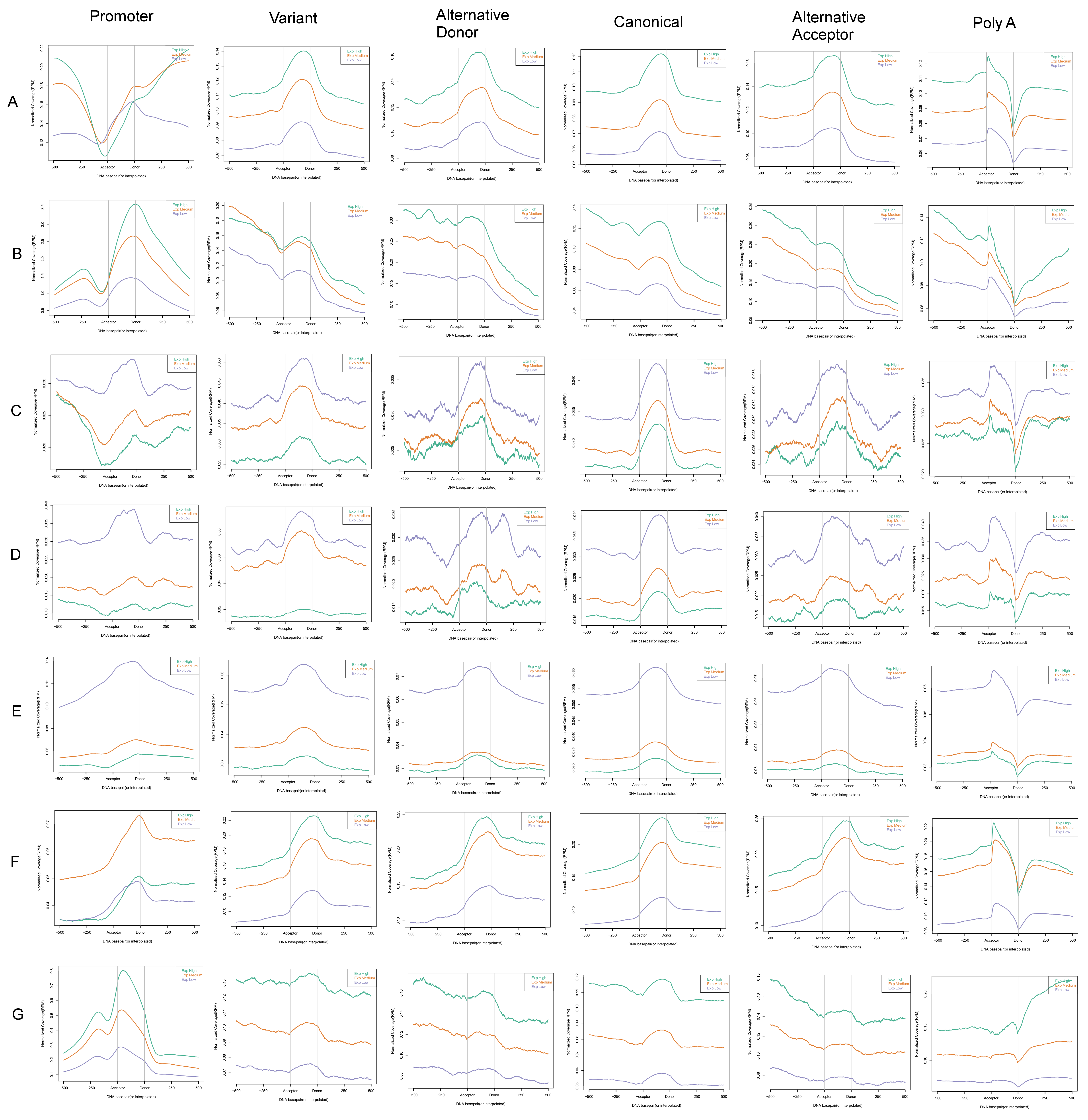

Supplement: Additional file 15: Figure S8 — Coverage plots for the seven marks at six different types of exons (see main text). The exons are further classified based on the RNA-seq RPKM of the corresponding transcript: 'High' (≥10), 'Medium' (≥1 and <10), and 'Low' (<1). (A) H3K4me1. (B) H3K4me3. (C) H3K9me2. (D) H3K9me3. (E) H3K27me3. (F) H3K36me3. (G) RNA pol II. [file gb-2014-15-4-r65-S15.tiff]

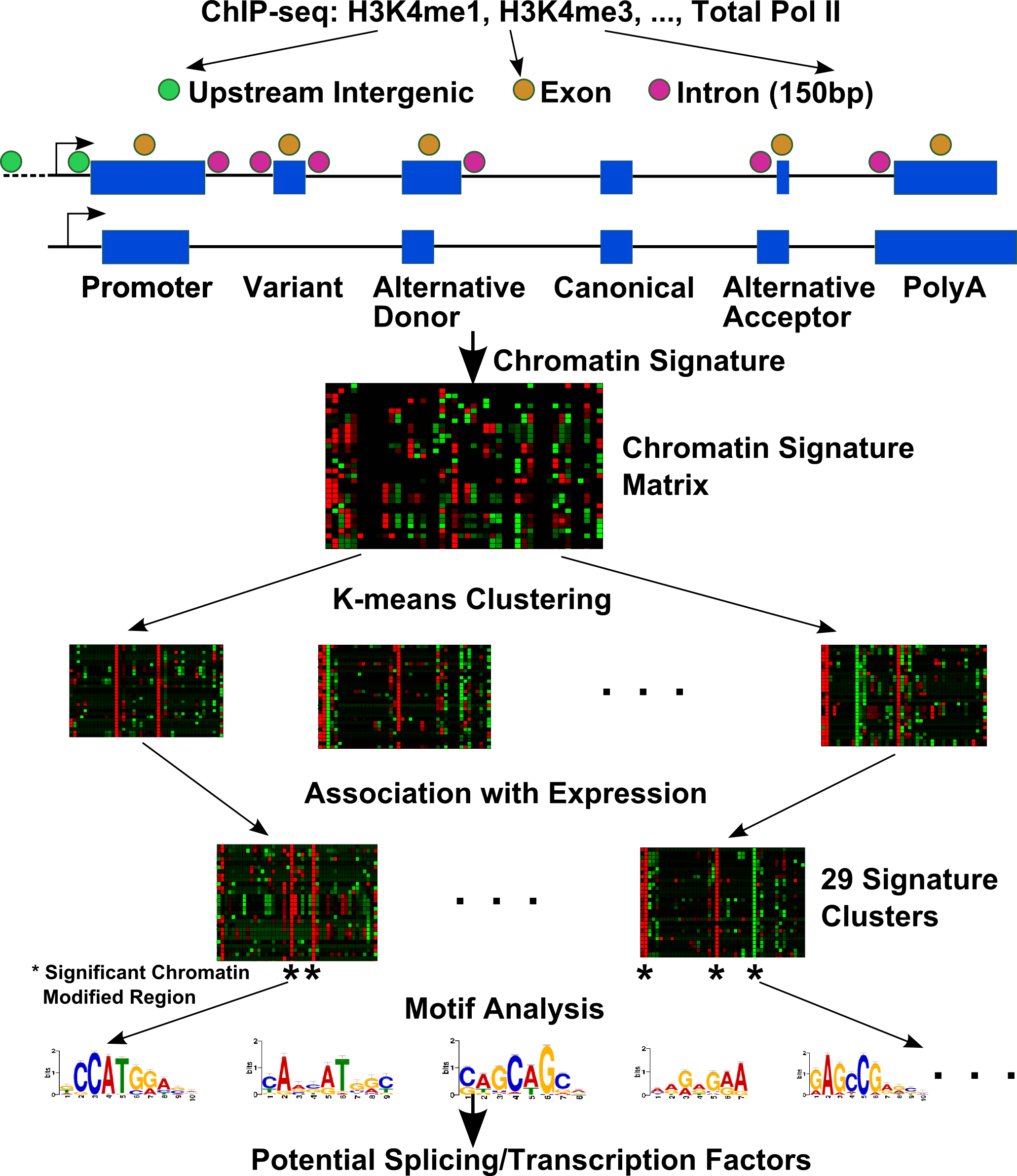

Supplement: Additional file 16: Figure S9 — Construction of cocaine-induced chromatin signatures. All chromatin signatures are put into a signature matrix with each row being a transcript and each column being the log fold change of each mark at each genomic region. K-means clustering was performed on the signature matrix to group transcripts into signature clusters that share common chromatin modification patterns. The regions that show significant chromatin changes were extracted to perform motif analysis to identify potential splicing and transcription factors. [file gb-2014-15-4-r65-S16.png]

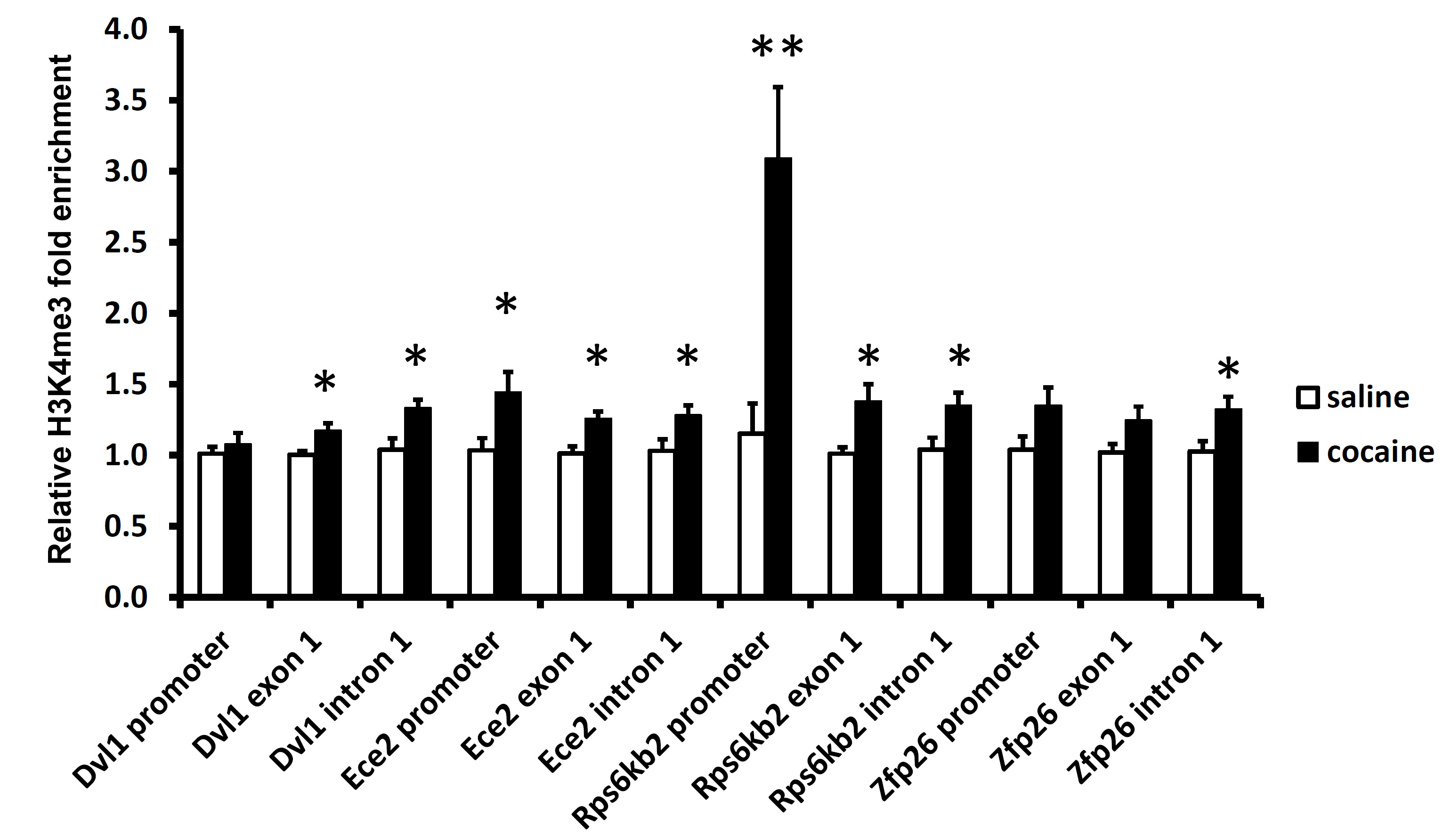

Supplement: Additional file 21: Figure S10 — Quantitative ChIP validation of cocaine-induced changes in H3K4me3 in NAc. A separate cohort of animals was used to validate ChIP-seq data. All genomic sites tested display the same direction of change with significance as seen with ChIP-seq. Error bars are mean ± standard error of the mean derived from 8 to 14 replicates per condition. *P < 0.05, **P < 0.01. [file gb-2014-15-4-r65-S21.jpeg]
