# Supplementary material for: Chronic cocaine-regulated epigenomic changes in mouse nucleus accumbens
Source: Genome Biol. 2014 Apr 22;15(4):R65. doi: 10.1186/gb-2014-15-4-r65 (PMC4073058; doi:10.1186/gb-2014-15-4-r65)
Supplement: Additional file 7 — Extended experimental procedures. [file gb-2014-15-4-r65-S7.docx]

Extended Experimental Procedures:

RNA-seq library prep

In brief, the poly-A containing mRNA was purified using poly-T oligo-attached magnetic beads. The mRNA was then fragmented into small pieces using divalent cations under elevated temperature. The cleaved RNA fragments were copied into first strand cDNA using reverse transcriptase and random primers. This was followed by second strand cDNA synthesis using DNA Polymerase I and RNaseH. These cDNA fragments went through an end repair process, the addition of a single ‘A’ base, and then ligation of the adapters. These products were gel purified and enriched with PCR to create the final cDNA libraries. The library constructs were run on the bioanalyzer to verify the size and concentration before sequencing on the Illumina HiSeq2000 machine at Mount Sinai’s Genomic Core facility.

ChIP-seq library prep

In brief, DNA fragment overhangs were converted into phosphorylated blunt ends, using T4 DNA polymerase, Klenow polymerase, and T4 polynucleotide kinase. An “A” base was then added to the DNA fragments to enable ligation to the adapters, which have a single “T” overhang. The constructed library was then run on a 2% agarose gel and size selected between 175-300 bp. Lastly, gel-extracted DNA was further enriched by PCR and run on a bioanalyzer to validate size distribution and concentration. All sequencing libraries were run on Illumina Hi-seq 2000 machines at Mount Sinai.

RNA-seq data analysis

The RNA-seq short reads were aligned against a reference gene database (Ensembl: Mus musculus, NCBIM37.62) using Tophat. The Cufflinks package was then used to perform differential analysis for various transcriptomic events with sequencing bias adjusted (--frag-bias-correct) and multiple alignment corrected (--multi-read-correct) to obtain a more accurate estimate of transcript abundance.

ChIP-seq data processing

The raw ChIP-seq data initially went through image analysis, base calling, quality filtering, and short read alignment using Illumina’s CASAVA pipeline. Only the uniquely aligned short reads were kept for further analysis. The numbers of uniquely aligned reads are listed in additional file 7. Next, we removed the potentially redundant short reads. The redundancy is caused by PCR amplification which represents an intrinsic limitation of second generation sequencing technology. To remove the PCR duplicates, we utilize the fact that, during the preparation of a ChIP-seq library, a sonicator breaks the whole genome almost uniformly so that most fragments come from unique positions (because a mammalian genome is ~2-3 billion bp long). Assuming a uniform breaking rate, we can use binomial distribution to determine the maximum allowed short reads at each position and strand, given the library and genome sizes with a significant p-value threshold (p=1E-5 used here). If the ChIP-seq library size is *L*, genome size is *G* and p-value cutoff is *p*, this can be done using the R function:

*qbinom(1-p, L/2, 0.5/G)*

The numbers of short reads after redundancy removal are listed in additional file 7. All the following analyses are based on these uniquely aligned and redundancy removed short reads.

ngs.plot: global visualization for NGS samples

Global visualization of a ChIP-seq sample is often useful to determine the enrichment of the protein of interest and to assess the data quality. We have therefore developed a program called ngs.plot (<https://code.google.com/p/ngsplot/>) to deal with this task as convenient as issuing a few command lines. Briefly, the alignment files are used to derive genomic coverage which is normalized to “aligned Reads Per Million mapped reads” (RPM) at single base resolution. A database is built into ngs.plot, which contains genomic coordinates of interesting biological features such as TSS, TES and genebody for a few model organisms. ngs.plot also allows input of customized genomic regions such as differential sites generated by diffReps. The coverage at those regions is then extracted and an average profile is calculated and plotted. For the regions of varied lengths, such as genebody, interpolation is used to fit the data points and then sampled at equal intervals. The three biological replicates are pooled to generate additional file 8.

Ranking functional terms by co-occurrence score

Functional enrichment analysis often generates a large number of enriched terms. When the analysis is repeated for multiple gene lists, such as the differential lists of the seven epigenomic marks, the list of enriched terms becomes too long to interpret clearly. In addition, many terms are often shared by multiple gene lists. The significance of a functional enrichment is expressed as a p-value. When –log10 function is applied to p-values, they can be added up to represent the overall enrichment of a functional term among multiple gene lists. The higher the sum, the more enriched the functional term is. We therefore define the co-occurrence score as the summation of –log10(p-value)’s for all gene lists under consideration. The co-occurrence score can then be used to rank functional terms in descending order. This co-occurrence score is applied to the enriched pathways among the ChIP-seq differential lists and the enriched functions and pathways among the 29 signature clusters in this study.

Enhancer definition by H3K4me1 peaks

Existence of enhancers is characterized in part by the enrichment of an H3K4me1 peak. Peak calling was done using HOMER [[1](#_ENREF_1)]. Because H3K4me1 is also involved in gene transcription or alternative splicing, we further removed any overlap with H3K4me3 peaks or exons and restricted the distance to any TSS to be >1kb to define an enhancer list. Finally, we defined 24,745 enhancers using the data merged from the two conditions. Based on the distance to TSS within 10kb, we found 8,827 protein coding transcripts to be within putative enhancers’ target zone. However, genome-wide association analysis [See additional file 17] did not report any correlation between chromatin modifications at the enhancers and the transcriptional change of their target transcripts. We further relaxed the distance cutoff to 1Mb and found 47,951 protein coding transcripts to be within putative enhancers’ target zone. However, still no correlation (data not shown) was found with the relaxed cutoff.

Defining and clustering chromatin signatures

Each exon is classified into six different types: “promoter”, “canonical”, “variant”, “alternative acceptor”, “alternative donor”, and “polyA”. “promoter” is the first exon at the 5’ end; “canonical” is the exon shared by other transcripts; “variant” is the exon skipped in other transcripts; “alternative acceptor” is the exon that contains the 5’ alternative boundary; “alternative donor” is the exon that contains the 3’ alternative boundary; and “polyA” is the last exon at the 3’ end. Each exon type represents a unique combination of exon-intron boundaries. Each exon is classified into the six categories by comparing its boundaries with exons in other transcripts of the same genes. This is achieved by exhaustively performing pairwise comparisons for all the transcripts within the same gene. For each transcript, all exons are sorted from 5’ to 3’ and defaulted to “canonical” except for the “promoter” and “polyA”. Each exon is then compared with exons in other transcripts and if an alternative boundary is found, it is classified as “altDonor” or “altAcceptor”; if it is overlapped with an intron, it is classified as “variant”. After classification, the genomic coordinates for all exons and neighboring intronic regions for all transcripts are output in BED [[2](#_ENREF_2)] format.

To accurately represent chromatin mark enrichment, ChIP-seq reads are shifted towards the center of the sequenced fragment based on the average fragment length of each library. Then the exon BED file is compared with the ChIP-seq alignment of the seven epigenomic marks and the number of intersections are counted using BEDTools [[3](#_ENREF_3)]. These count files are analyzed again by summing up the counts for the same exon type for each transcript. A cutoff of 30 is used to filter low count regions. The counts are first normalized to corresponding library sizes and then log2 fold changes are calculated between the two conditions. These log2 fold changes are formatted as chromatin signatures and all signatures are assembled into a data matrix. In this way, we can create a unified model to represent the epigenomic changes of all transcripts so that they can be used for clustering analysis. Although this model may cover the changes of an individual exon, it can also capture the overall changes of the exons that belong to the same type. After the groups of transcripts with similar chromatin patterns are found, each individual exon is further analyzed to see if it is consistent with the overall pattern before motif analysis.

In the signature matrix, each row represents a transcript and each column represents a mark-region combination. Genome-wide association is performed on each column to test its correlation with transcriptional change. This is done by first determining the transcripts that show significant chromatin modifications at a particular region. The mean and standard deviation (SD) for each column are calculated. A transcript is considered to be significantly regulated if its value is at least 1.5 SD deviated from the column mean. Therefore, two groups of transcripts that show increased and decreased chromatin binding are defined for each column. The enrichment of transcriptional change for the two groups is evaluated using Fisher’s exact test. This gives us four different combinations in the form of chromatin-expression pairs: up-up, up-down, down-up, down-down. A column is considered significant if any of the four combinations passes a cutoff of FDR<10%. We did not find significant correlation for the enhancer regions for all seven marks [See additional file 17]. The enhancer regions are removed altogether and the genome-wide association is repeated using the same cutoff. 43 columns are kept after this. Each transcript is also evaluated for chromatin modification and is filtered if none of the 43 columns contains a value that is 1.5 SD away from the column mean. This process reduces the number of transcripts to ~33,000.

K-means clustering is performed on the filtered signature matrix to identify co-expressed patterns using the "Hartigan-Wong" algorithm. The number of clusters is set to 300 and the process is restarted 10,000 times to approximate the best solution. Each time the program is allowed to iterate 1,000 times or the sum of squares converges (tolerance=1E-6), whichever comes first. The resulting clusters range from 21 to 766 transcripts in size. All signature clusters are then compared with the list of transcripts that shown increased or decreased expression. Fisher’s exact test is used to evaluate the statistical significance of the enrichment of transcriptional change using the genome as background. An FDR<25% is chosen as cutoff to define significant signature clusters, which corresponds to P<0.01 and <0.02 for increased and decreased transcription, respectively.

Motif analysis for signature clusters

Among the 29 signature clusters, each exon or intronic region is only used for motif analysis when its averaged log2 fold change is significantly deviated from zero (P<1E-10, one-group T-test) over the three biological replicates. The individual change also has to be consistent with the corresponding cluster’s change. Multiple exons or intronic regions may be identified for each signature cluster. The DNA sequences for each significant region are extracted and output in FASTA files. MEME is used to identify enriched motifs with its default significance cutoff and up to 20 motifs are output. The genic (exon + intronic 150bp) and upstream intergenic regions are treated slightly differently. For genic regions, motif length is set between 4 and 10bp, and only the forward strand is considered. For upstream intergenic regions, motif length is set between 6 and 20bp, and both forward and reverse complemented strands are considered. The motif length choice is motivated by the fact that splicing factor binding motif tends to be shorter and more degenerated.

The enriched motifs may be represented more than once among the signature clusters because a transcription/splicing factor can control multiple groups of transcripts. Bayesian motif clustering [[4](#_ENREF_4)] is performed to group the motifs that are similar with each other. This generates a list of uniquely represented motifs which are then compared with motif databases by TOMTOM [[5](#_ENREF_5)] to identify known protein regulators. The mouse UniPROBE (http://meme.nbcr.net/downloads/databases/) database is used to identify transcription factors. The UniPROBE and the RBPDB [[6](#_ENREF_6)] are used to identify splicing factors.

Nuclear protein isolation protocol

NAc punch dissections were homogenized using a Dounce homogenizer in 1 ml of homogenizing buffer (10 mM Tris-HCl buffer, pH 7.5, 0.32M sucrose, 1 mM EDTA, 1 mM EGTA, 5 mM DTT, and protease inhibitor), followed by centrifugation at 1000 ×g for 10 min. Pellets were resuspended in 1 ml of homogenizing buffer containing 0.5% Nonidet P-40, followed by centrifugation at 1000 × g for 10 min to obtain nuclear fractions. These nuclear fractions were resuspended for Western blot analysis.

Reference

1. Heinz S, Benner C, Spann N, Bertolino E, Lin YC, Laslo P, Cheng JX, Murre C, Singh H, Glass CK: **Simple Combinations of Lineage-Determining Transcription Factors Prime cis-Regulatory Elements Required for Macrophage and B Cell Identities.** *Molecular Cell* 2010, **38:**576-589.

2. **BED Format (**[http://genome.ucsc.edu/FAQ/FAQformat - format1](http://genome.ucsc.edu/FAQ/FAQformat#format1)**)**

3. Quinlan AR, Hall IM: **BEDTools: a flexible suite of utilities for comparing genomic features.** *Bioinformatics* 2010, **26:**841-842.

4. Jensen ST, Liu JS: **Bayesian clustering of transcription factor binding motifs.** *Journal of the American Statistical Association* 2008, **103:**188-200.

5. Tanaka E, Bailey T, Grant CE, Noble WS, Keich U: **Improved similarity scores for comparing motifs.** *Bioinformatics* 2011, **27:**1603-1609.

6. Hughes TR, Cook KB, Kazan H, Zuberi K, Morris Q: **RBPDB: a database of RNA-binding specificities.** *Nucleic Acids Research* 2011, **39:**D301-D308.
