# Supplementary material for: Chronic cocaine-regulated epigenomic changes in mouse nucleus accumbens
Source: Genome Biol. 2014 Apr 22;15(4):R65. doi: 10.1186/gb-2014-15-4-r65 (PMC4073058; doi:10.1186/gb-2014-15-4-r65)
Supplement: Additional file 25: Table S12 — Quality control of RNA-seq data. RPKM values are shown for the three saline samples from the acute experiment and from the chronic experiment. MSN-enriched genes: genes known from previous studies to be enriched in striatal (including NAc) medium spiny neurons. MSN-depleted genes: genes encoding related neurotransmitter and neuropeptide system products known from previous studies to be expressed at low levels in NAc. Choroid plexus-enriched genes: genes known from previous studies to be enriched in choroid plexus, although many of these genes are also known to be expressed in neurons. [file gb-2014-15-4-r65-S25.pdf]

Table S12, Quality Control of RNA-Seq Data.

	Gene Symbol	S1	S2	S3	Mean acute	S1	S2	S3	Mean chronic	Notes[1]
MSN-enriched genes	Adcy5	140.3	155.4	140.4	145.3	179.4	181.4	168.0	176.3	
	Arpp19	238.7	287.8	273.5	266.7	269.1	301.8	240.6	270.5	
	Cartpt	30.7	29.8	25.1	28.5	35.8	29.0	46.2	37.0	
	Chrm1	19.9	20.0	20.8	20.2	24.2	25.2	23.5	24.3	Higher in olfactory tubercle
	Gnb5	86.8	93.4	91.5	90.6	96.0	98.1	94.4	96.2	
	Gng7	290.1	339.5	296.0	308.5	367.4	366.0	368.4	367.3	
	Mchr1	18.3	20.4	17.0	18.6	19.3	18.6	20.0	19.3	Higher in dorsal striatum
	Npy	62.7	54.9	59.3	59.0	32.1	35.7	29.3	32.4	
	Pde10a	118.4	137.3	126.7	127.5	215.5	227.2	220.5	221.1	
	Pdyn	81.4	89.6	81.4	84.1	106.0	100.8	119.8	108.8	
	Penk	738.0	876.5	794.7	803.1	738.3	781.9	744.0	754.8	
	Ppp1r1b	592.7	635.5	555.0	594.4	552.8	590.0	535.2	559.3	
	Ptpn5	127.3	137.2	128.5	131.0	181.0	171.8	177.3	176.7	
	Rasgrp2	75.6	85.7	87.4	82.9	102.7	106.1	102.1	103.6	
	Rgs9	117.6	127.6	119.7	121.6	160.0	161.4	167.5	162.9	
	Tac1	204.7	227.7	196.3	209.6	164.0	168.6	184.6	172.4	
MSN-depleted genes	Adcy10	0.0	0.0	0.0	0.0	0.0	0.0	0.0	0.0	Higher in dorsal striatum and olfactory tubercle
	Bdnf	1.2	1.0	2.2	1.5	0.3	0.5	0.3	0.4	Higher in olfactory tubercle
	Chrm2	1.9	1.7	2.2	1.9	1.6	1.8	1.4	1.6	Higher in olfactory tubercle and septum; also expressed in choroid plexus
	Chrm3	3.5	2.9	4.3	3.6	1.6	2.5	1.3	1.8	Higher in olfactory tubercle
	Chrna2	0.1	0.1	0.1	0.1	0.1	0.1	0.1	0.1	Higher in olfactory tubercle and septum
	Chrna4	5.5	4.0	6.1	5.2	3.6	4.7	3.4	3.9	Higher in

										lateral cortex
	Chrna7	4.3	4.0	4.5	4.3	4.6	4.7	4.3	4.5	Higher in olfactory tubercle
	Gfra3	0.0	0.0	0.0	0.0	0.0	0.0	0.0	0.0	
	Hcrt	0.0	0.0	0.0	0.0	0.1	0.0	0.1	0.1	
	Pmch	0.1	0.1	0.0	0.1	0.1	0.1	0.0	0.1	Higher in dorsal striatum
	Slc6a3	0.2	0.1	0.2	0.2	0.1	0.1	0.1	0.1	
	Slc6a4	0.0	0.1	0.0	0.0	0.0	0.0	0.0	0.0	
	Th	4.2	4.4	4.3	4.3	3.8	4.3	3.8	3.9	
	Tph1	0.0	0.1	0.0	0.1	0.0	0.0	0.0	0.0	
Choroid plexus-enriched genes[2]	Apoa2	0.5	0.2	0.3	0.3	0.3	0.3	0.3	0.3	
	Aqp1	0.1	0.1	0.1	0.1	0.1	1.0	0.1	0.4	
	Atp4a	0.1	0.1	0.1	0.1	0.1	0.0	0.1	0.1	
	Folr1	0.1	0.1	0.1	0.1	0.1	2.8	0.5	1.1	
	Hspb1	2.1	2.9	3.2	2.7	1.0	0.9	0.9	0.9	
	Klk4	0.0	0.0	0.0	0.0	0.0	0.0	0.0	0.0	
	Mmp14	3.3	3.3	2.8	3.1	2.7	2.7	2.9	2.8	
	Slc16a12	0.4	0.4	0.4	0.4	0.4	0.5	0.4	0.5	
	Slc19a1	5.8	5.4	5.0	5.4	6.5	6.1	5.5	6.0	
	Slc5a5	2.6	2.0	2.9	2.5	0.9	1.2	0.7	0.9	
	Ttr	1.4	3.5	5.5	3.5	5.1	222.2	1.7	76.3	

[1]Genes known to be expressed at higher levels in brain areas in close proximity to NAc.

[2]Only 10 representative genes are shown from the ~100 mouse choroid plexus enriched gene list compared to striatum [76].
